# Mucolytic Effectiveness of Tyloxapol in Chronic Obstructive Pulmonary Disease - A Double-Blind, Randomized Controlled Trial

**DOI:** 10.1371/journal.pone.0156999

**Published:** 2016-06-16

**Authors:** Martin Koppitz, Charlotte Eschenburg, Emilia Salzmann, Martin Rosewich, Ralf Schubert, Stefan Zielen

**Affiliations:** 1 Department for Children and Adolescents, Division for Allergology, Pneumology and Cystic Fibrosis, University Hospital Goethe University, Frankfurt am Main, Germany; 2 Department for Children and Adolescents, Division for Stem Cell Transplantation and Immunology, University Hospital Goethe University, Frankfurt am Main, Germany; University of Texas Health Science Center at Tyler, UNITED STATES

## Abstract

**Objective:**

Mucoactive drugs should increase the ability to expectorate sputum and, ideally, have anti-inflammatory properties. The aim of the study was to evaluate the mucolytic activity of Tyloxapol compared to saline (0.9%) in COPD.

**Design:**

A randomized, placebo-controlled, double-blinded crossover, clinical trial was carried out. Patients were randomly assigned to either inhale 5 ml Tyloxapol 1% or saline 0.9% solution three times daily for 3 weeks and vice versa for another 3 weeks. 28 patients (18 male, 10 female, 47 to 73 years old, median age 63.50) were screened, 21 were treated and 19 patients completed the study per protocol.

**Results:**

A comparison of the two treatment phases showed that the primary endpoint sputum weight was statistically significant higher when patients inhaled Tyloxapol (mean 4.03 g, 95% CI: 2.34–5.73 g at week 3) compared to saline (mean 2.63 g, 95% CI: 1.73–3.53 g at week 3). The p-value at three weeks of treatment was 0.041 between treatment arms. Sputum cells decreased during the Tyloxapol treatment after 3 weeks, indicating that Tyloxapol might have some anti-neutrophilic properties. Lung function parameters (FVC, FEV_1_, RV, and RV/TLC) remained stable during the study, and no treatment effect was shown. Interestingly, there was a mean increase in all inflammatory cytokines (IL-1β, IL-6, and IL-8) during the saline treatment from day 1 to week 3, whereas during the Tyloxapol treatment, all cytokines decreased. Due to the small sample size and the large individual variation in sputum cytokines, these differences were not significant. However, analyses confirmed that Tyloxapol has significant anti-inflammatory properties in vitro. Despite the high number of inhalations (more than 1000), only 27 adverse events (20 during the Tyloxapol and seven during saline) were recorded. Eleven patients experienced AEs under Tyloxapol and six under saline treatment, which indicates that inhalation of saline or Tyloxapol is a very safe procedure.

**Conclusion:**

Our study demonstrated that inhalation of Tyloxapol by patients with COPD is safe and superior to saline and has some anti-inflammatory effects.

**Trial Registration:**

ClinicalTrials.gov NCT02515799

## Introduction

In chronic bronchitis, mucus hypersecretion is the key presenting symptom, contributes to airflow obstruction and has been the subject of debate for a long time [1]. According to Fletcher and Peto’s seminal paper mucus hypersecretion “the hypersecretory disorder” did not seem to correlate with chronic airway obstruction [2], but this finding was later challenged [3][4]. By the 1950s, it was known that tobacco smoking was associated with chronic cough and sputum production and that smokers who developed chronic bronchitis had impaired lung defences, a condition favouring bacterial colonization and infection of the lower airways [5]. Biopsies have shown that excess mucus production, which defines chronic bronchitis, is associated with enlarged bronchial glands and that an approximate relationship exists between the presence of chronic bronchitis and emphysema. It is well established that chronic mucus hypersecretion is significant and consistently associated with both a decrease in forced expiratory volume (FEV_1_) and an increase in subsequent hospitalization [3, 4, 6].

Drugs that affect airway secretion have existed for many years. Based on their potential mechanism of action, drugs can be classified as expectorants, mucoregulators, mucolytics or mucokinetics. However, many drugs exhibit overlapping effects. A large number of studies have been performed on the use of mucolytic drugs in the treatment of chronic bronchitis and chronic obstructive bronchitis (COPD), and the outcomes have been reviewed in several meta-analyses [7, 8]. Interestingly, a recent Cochrane review concluded that the treatment of COPD with mucolytics may produce a small reduction in acute exacerbations and a small effect on overall quality of life [9]. Mucolytics are well tolerated, and there are fewer adverse events than with placebo [7]. Current guidelines however do not recommend regular use of mucolytics even in the chronic bronchitis phenotype of COPD. Beyond anticholinergics with a small effect size on mucus hypersecretion, a frequent complaint of CB and COPD patients, as yet no drugs are available on the market reducing mucus hypersecretion [10]. Still there is an unmet need for an ideal drug to ameliorate airway secretions in COPD.

The mucolytic agent Tyloxapol has been used therapeutically for over 50 years and has proven to be well tolerated during this time [11–15]. Side-effects in the form of hypersensitivity reactions have only occurred very rarely. Tyloxapol is a polymeric covalent compound with multiple mucolytic actions. Tyloxapol influences the respiratory system by the following four different action mechanisms: secretolytic action, reduction of surface tension, dissolution of coatings and down-regulation of inflammation.

Several studies have shown that small quantities of Tyloxapol applied as an aerosol liquefy sputum [16, 17]. The viscosity of sputum is reduced by 10% to 20% according to rotational viscosimetry measurements [18]. In a double-blind crossover study of 20 patients with COPD, Paez et al. compared sodium chloride solution, distilled water, and Tyloxapol with regard to their ability to act secretolytically. Interestingly, Tyloxapol led to a significant increase in sputum volume and sputum dry weight (by 0.84 g/h; 95% CI 0.15–1.54 g/h, n = 7) compared with distilled water [17].

Farber et al. showed that Tyloxapol also penetrates the mucous wall and dissolves viscous and dried secretions, thus enabling increased ciliary activity in the respiratory tract [19].

Tyloxapol inhibits activation of the transcription factor nuclear factor-kappa B (NF-kappa B) and reduces resting secretion of the cytokine interleukin-8 (IL-8) in cultured human monocytes. Tyloxapol also inhibits pro-inflammatory cytokines such as lipopolysaccharide (LPS)-stimulated release of tumour necrosis factor-alpha (TNF-α), IL-1 beta, IL-6, IL-8, and granulocyte-macrophage colony-stimulating factor (GM-CSF), as well as the eicosanoids thromboxane A2 and leukotriene B4 [20].

Although the mechanism of Tyloxapol has been well described, and there is a long-standing basis for its clinical usefulness, there are no randomized, double-blind, placebo-controlled trials available that demonstrate the superiority of Tyloxapol vs. saline. The aim of the present study was to assess the effects of the mucolytic agent, Tyloxapol compared to saline (0.9%) in patients with COPD.

## Methods

### Study design

The study was a monocentric, randomized, double-blind, crossover, placebo-controlled study of COPD patients. The study was approved by the Ethics Committee of Frankfurt Goethe University at the 14^th^ of July 2014 and sponsored by bene-Arzneimittel GmbH (Munich, Germany). The human experimentation guidelines of Good Clinical Practice, the German Drug Act and the declaration of Helsinki/Hong Kong were followed in the conduct of the clinical research. The ClinicalTrials.gov Identifier is NCT02515799. The registration of the trial was send to Clinicaltrials.gov before the trial was started, but unfortunately the trial could not be uploaded right in time. This was in part due to the fact that the trial was planned as an investigator initiated study by the principal investigator (PI) but during the application process it changed to a full sponsored trial. Full sponsored trials cannot be uploaded and registered by the PI. Sponsored trials have to be uploaded by the sponsor. The PI can ensure that the trial design, results and analyses of data were not affected by the delay of study registration.

All patients gave their written informed consent prior to inclusion in the study. The recruitment of the study was started at the 4^th^ of August 2014 and the last patient out was at the 22^nd^ of December 2014.

### Dosage and treatment schedule

In the study, we used Tacholiquin^®^ (bene-Arzneimittel GmbH, München Germany) containing 1% Tyloxapol, 5% glycerine and 2% sodium hydrogen carbonate in a sterile aqueous solution and saline 0.9% solution. The study medication was inhaled using the PARI Turbo BOY SX^®^ (PARI GmbH, Starnberg, Germany) three times daily. The preparation was available in a 5-ml syringe and was stable for 4 weeks. Matching syringes with 5 ml 0.9% saline were used as placebo. The sequentially numbered containers were delivered by the pharmacy Central Apotheke (Steinbach, Germany). The syringes did not differ in appearance (concerning form, weight, color, texture of content, etc.), to ensure patient and investigator blinding.

**Fig 1:**
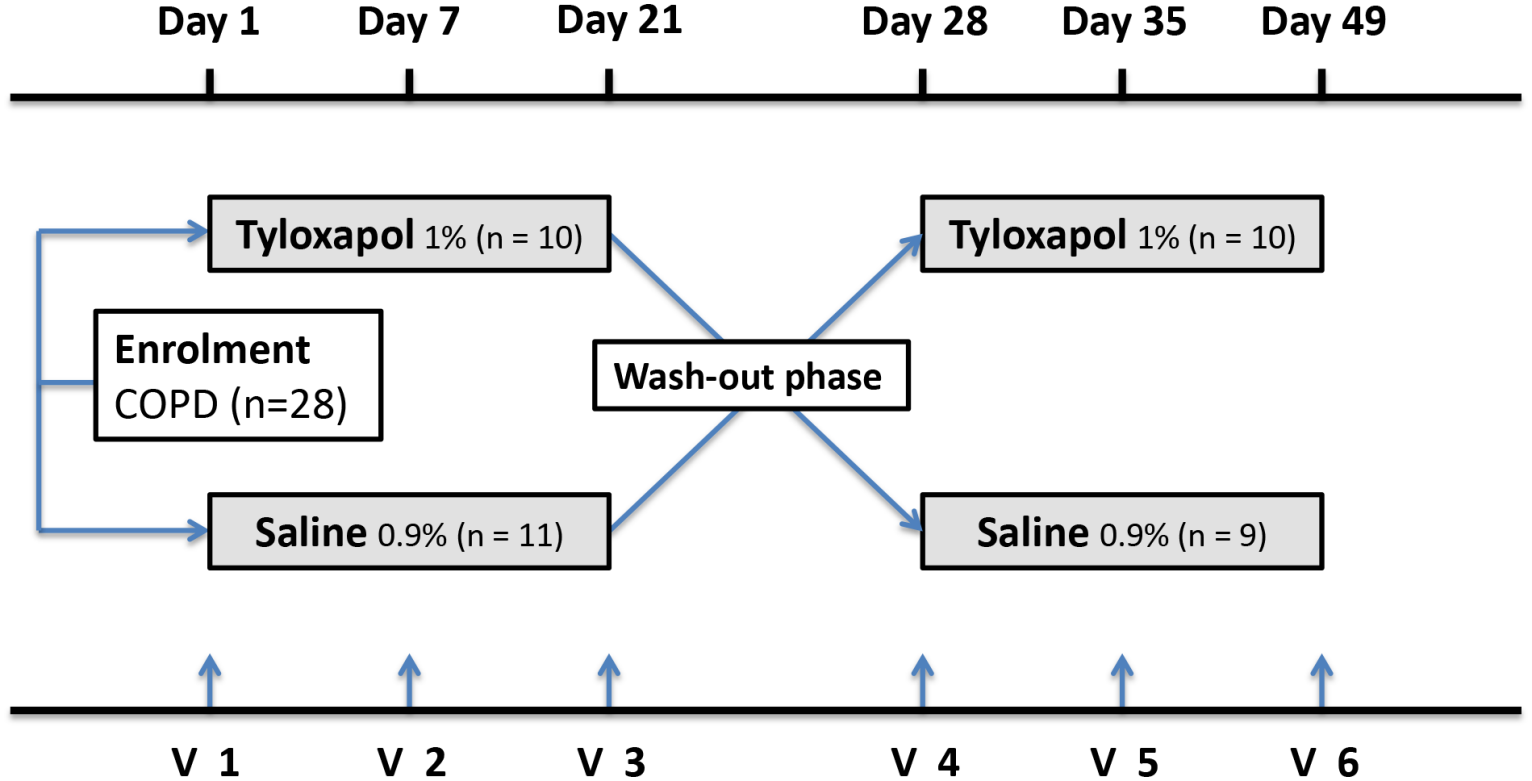
Study design. Phase A patients were treated with either Tyloxapol or saline for 21 days at three inhalations a day. After a wash-out phase of 7 days, Phase B started. Patients were switched to the other medication for another 21 days.

The dosage regimen involved three inhalations per day of 5-ml solutions via nebulizer over 21 consecutive days. Patients were randomized to start with either the Tyloxapol or saline 0.9% solution in treatment phase A. After treatment phase A, patients received the other solution in treatment phase B after 7 days of wash-out (see Fig 1).

### Patients

Twenty-eight patients were initially enrolled in the study (Table 1). Patients were recruited from own database and by public advertisements. The study was performed at the study centre Medaimun GmbH Frankfurt, Germany. After informed consent, a physical examination was performed at each visit. Patients with a diagnosis of COPD and a smoking history of more than 10 pack-years were included. Further criteria for inclusion were as follows: age between 40 and 85 years, FEV_1_ ≤ 85% and FEV_1_/FVC ≤ 0.7, COPD Assessment Test (CAT) ≥ 10, complaining chronic coughing or sputum expectoration several times a week as well as no exacerbation four weeks prior to visit 1.

**Table 1:** Clinical and demographic characteristics of patients. Values are the median and range.

	Enrolment	Treated	Per Protocol
	n = 28	n = 21	n = 19
Age (years)	**63.5** (47–73)	**61** (47–71)	**60** (47–71)
Sex (male in %)	**64.29**	**71.43**	**68.42**
Body size (m)	**1.75** (1.59–1.91)	**1.78** (1.61–1.91)	**1.77** (1.61–1.91)
Body weight (kg)	**81.5** (56.5–105)	**86** (56.5–103)	**86** (56.5–103)
FVC (%)	**76** (35–99)	**77** (39–99)	**78** (39–99)
FEV_1_ (%)	**52.5** (24–82)	**53** (26–82)	**56** (26–82)
FEV_1_ (%) After Salbutamol 400 μg	**56.5** (26–82)	**59** (32–82)	**61** (37–82)
Pack years (n)	**45** (15–100)	**47** (15–100)	**47** (15–100)
Active smokers (%)	**64.29**	**61.91**	**63.16**
CAT (points)	**22.5** (8–33)	**22** (10–32)	**22** (10–32)

The reasons for exclusion were as follows: acute upper or lower respiratory infection requiring antibiotics or antiviral medication within 4 weeks prior to visit 1; clinically important pulmonary disease other than COPD (e.g., clinically significant bronchiectasis, pulmonary fibrosis, cystic fibrosis, lung cancer, alpha 1 anti-trypsin deficiency and primary ciliary dyskinesia) or other diagnosed diseases that are associated with elevated peripheral eosinophil counts; long term oxygen therapy; documented unstable ischaemic heart disease, arrhythmia, cardiomyopathy, heart failure, renal failure, uncontrolled hypertension or any other relevant disease; treatment with systemic corticosteroids and/or immunosuppressive drugs; or a known history of anaphylaxis to Tyloxapol.

Clinical symptoms were assessed by the COPD Assessment Test (CAT) at each visit. Any improvements in dyspnoea were assessed via the Baseline and Transition Dyspnoea Index (BDI/TDI) throughout each treatment phase. Quality of life measures were made using the St George’s respiratory Quality of Life Questionnaire (SGRQ) a weighted score given in percent, with higher scores indicating more limitations. Self-completed questionnaires were performed before and after each treatment phase. Patients were asked to report their subjective view of mucous congestion and ease of sputum expectoration using a visual analogue scale (Likert 1 and 2) after sputum expectoration at each visit. Patients kept a diary during the study to record all adverse events (AE) and concomitant medication during the study.

### Pulmonary function test

First, subjects performed a lung function analysis, then inhaled 400 μg of Salbutamol, and the lung function was reassessed after 15 minutes. A whole-body plethysmography was performed according to Criee et al [21]. The following variables were documented: FVC [L, % pred], FEV_1_ [L, % pred], MEF25 [L, % pred], FEV_1_/FVC (Tiffeneau-Index, % pred), RV [% pred] and RV/TLC [% pred].

### Sputum collection and processing

Subjects inhaled nebulised study medication Tyloxapol or isotonic saline. Afterwards, the patients were asked to expectorate. During this procedure, it was important to flush and clean the nose to avoid epithelial cells in the samples. The subjects underwent two subsequent lung function analyses at 30 and 60 min after the nebulisation. Sputum was processed at 4°C within 2 hours of collection. First, the weight of the whole sputum sample was measured (pre-selected sputum). Then, sputum plugs were selected by using forceps as previously described (selected sputum) [22]. The selected sputum plugs were processed with 0.1% dithiothreitol [23]. Afterwards, 2x weight/volume of phosphate-buffered saline (PBS) was added. Samples were filtered through a 70-μm mesh and centrifuged for 10 minutes at 790 x g to remove the cells. Supernatants were stored at -80°C until analysis with a cytometric bead array.

For sputum cell counts, cytospins were prepared. Each stage was processed using Pappenheim's stain and fixed with Eukitt. Specimens containing levels of squamous epithelial cells less than 10% of the total inflammatory cell count were considered adequate. At least 400 inflammatory cells were microscopically counted for each stage [24]. Neutrophils, eosinophils, lymphocytes, basophils and macrophages were counted, and their percentage of the total cell count was calculated.

### Macrophage activation test (MAT)

The anti-inflammatory effect of Tyloxapol was measured by cytokine release after LPS-stimulation of whole blood cells. Briefly, EDTA-blood samples were diluted 1:10 with RPMI 1640 medium and stimulated with 5 ng/ml LPS from E. coli 026:B6 (Sigma-Aldrich Pharmaceuticals, St. Louis, Missouri, USA) in the presence of 0, 0.001, 0.01, 0.1 and 1.0 mg/ml Tyloxapol [25]. After a 24-hr incubation period at 37°C and 5% CO2, supernatants were harvested and stored at -80°C until further use.

### Cell viability

Whole blood cells were treated with LPS in the presence of of 0, 0.001, 0.01, 0.1 and 1.0 mg/ml Tyloxapol or 3% and 7% NaCl for 24 hrs at 37°C and 5% CO2. 7-Amino-actinomycin D (7-AAD) staining was used for the detection of dead cells. Therefore, samples were stained with 7AAD solution (Calbiochem, San Diego, CA) at 4°C protected from light for 20 minutes and analysed by flow cytometry (FACSVerse, Becton Dickinson, San Jose, CA, USA).

### Cytometric bead array

The concentrations of IL-1β, IL-6, IL-8 and RANTES were determined in sputum samples and cell culture supernatants (MAT) using the BD^™^ CBA Flex Set System (BD Bioscience-PharMingen, San Diego, CA, USA). Each BD^™^ CBA Flex Set contained one bead population with a distinct fluorescence intensity as well as the appropriate phycoerythrin (PE) detection reagent and standard. The tests were performed according to the manufacturer’s instructions. For analyses of the cytokines in sputum, we added the same concentration of DTT (0.025%) as in the sputum supernatant to the standard curve and enzyme immunoassay buffer as described recently [26].

### Sample size calculation

The primary outcome measure was change in pre-selection sputum weight after three weeks of treatment as difference between Tyloxapol and saline. The calculated sample size of 20 patients is based on an expected difference for Tacholiquin^®^ in sputum weight of about 1.2 g corresponding to about 40% of that expected for saline with a standard deviation of s = 1.7 g roughly corresponding to a correlation between sputum weights of ρ = 0.75. Then a sample size of 20 achieves a power of 80%”. This calculation was higher than the number of patients examined by Paez et al (n = 10) and consistent with an own previous study in COPD [17, 26]. In addition the cross-over design minimizes the interindividual differences in sputum expectoration of COPD patients. To account for up to 20% of recruited patients not being able to provide an adequate induced sputum sample, we set an initial enrolment goal of 30 patients.

Treatment assignment eligible patients were assigned in blocks (n = 10) to ensure that half of them started with inhalation of either saline or Tyloxapol to minimize carry over effects of treatment in the cross-over design.

### Statistical analysis

The aim of the study was to compare Tyloxapol and saline regarding their ability to promote the discharge of mucus in patients with COPD.

The primary outcome measure was change in pre-selection sputum weight after three weeks of treatment as difference between Tyloxapol and saline.

The explorative secondary outcome measures were: symptom scores (CAT, BDI, TDI and SGRQ), ease of sputum by Likert-scale, changes in lung function parameter (FVC, FEV_1_, RV and RV/TLC), cells and biomarkers (IL-1β, IL-6 and IL-8) in sputum. In addition acute changes of pre- and selected sputum weight at every visit was exploratively analyzed as described for the primary outcome measure.

Values are given as median and 95% confidence interval (CI) if not indicated differently.

Safety was assessed by monitoring of symptom scores and rescue medication, lung function FEV_1_ and adverse events (AEs).

The data analysis was performed using either GraphPad Prism 5 (GraphPad Software, La Jolla, USA) or R software for statistical computing, version 3.1.3, with the nlme package for longitudinal analysis. Comparisons between the two groups were analysed using the Mann-Whitney U-test or Student’s t-Test after the Shapiro-Wilk test of normality was applied. In addition smoking status was analysed as a possible confounder. For the longitudinal analysis, we applied a mixed-effect model with linear spline regression to determine the dynamic effect of Tyloxapol compared to placebo on sputum secretion. The fixed effect was the treatment, and we considered the subject to be the random effect. For the AEs odds ratio and confidence interval (CI) were calculated by median-unbiased estimation and p-value corresponding to Exact Fisher Test. All tests were two-sided, with a significance level of α =  5% and accounting appropriately for dependencies between samples from the same test persons and missing values. There was no adjustment for multiple testing necessary.

## Results

### Characteristics of the study population

**Fig 2:**
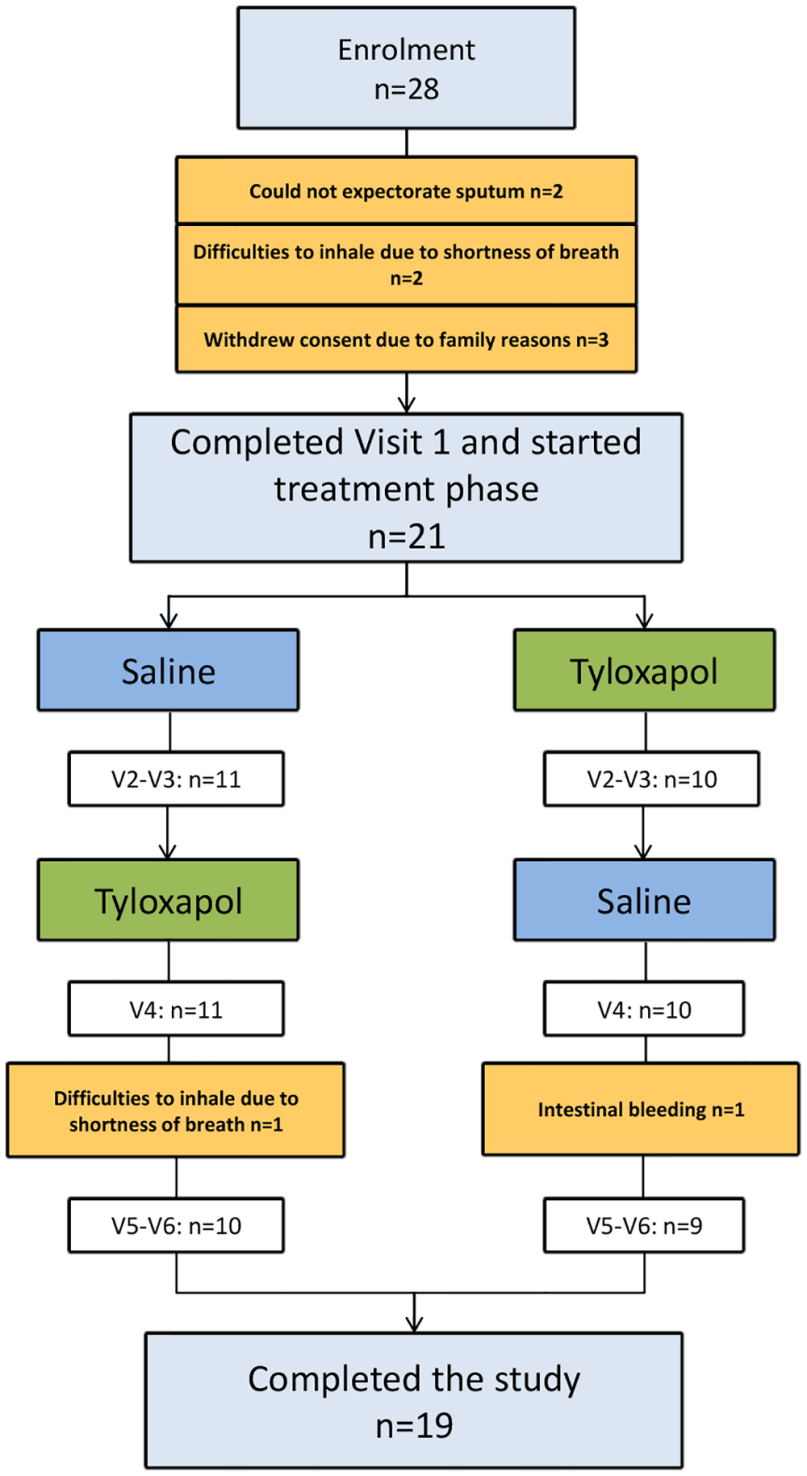
Patient flow chart. 28 patients were initially enrolled, 21 completed visit 1 and started treatment phase, 19 finished the study, 2 dropped out because of the following: intestinal bleeding (n = 1) and difficulties to inhale due to shortness of breath (n = 1).

Twenty-eight patients (18 male, 10 female, 47 to 73 years old, median age 63.50 years) were initially enrolled in the study (Table 1). During enrolment 7 patients could not start with the planned treatment phase due to several reasons (Fig 2). Nineteen patients completed the study per protocol. The enrolled population (n = 28) did not differ significantly from the treated (n = 21) and per protocol population (n = 19). Twelve of the 19 patients were active smokers, and the mean CAT was 21.1 points.

Five of the 19 patients per protocol were not treated with a prophylactic. Of the others, three received a monotherapy (LAMA or ICS), eight were treated with a double therapy (ICS+LABA or ICS+LAMA or LABA+LAMA), and three patients used a triple therapy (ICS+LAMA+LABA). Furthermore, two of the 19 patients were also treated with theophylline.

### Sputum weight

**Fig 3:**
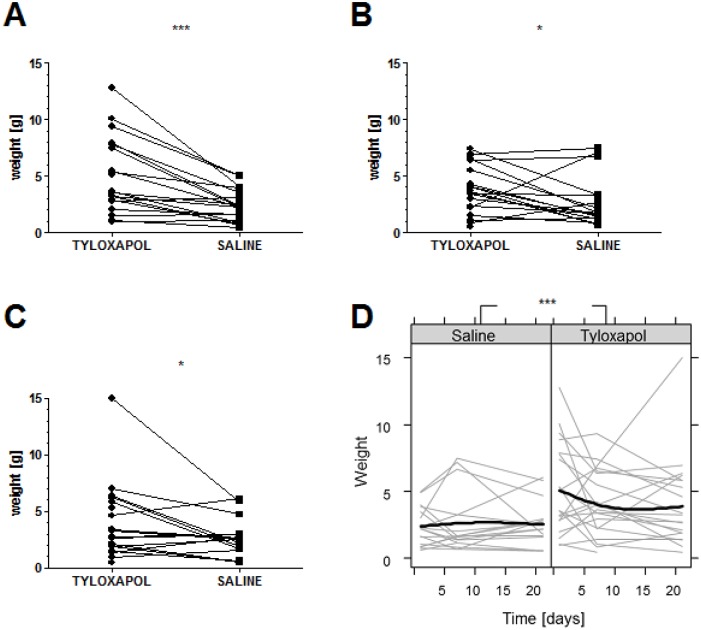
Sputum weight before processing comparing Tyloxapol and saline. The figures illustrate (A-C) the sputum weight of patients treated with Tyloxapol and saline before further processing at (A) day 1 (n = 19 vs. 18), (B) 1 week (n = 19 vs. 17) and (C) 3 weeks of inhalation (n = 18 vs. 16), and (D) the fitted mixed linear model for the weight of sputum secretion [g]. The black lines represent the predicted mean of each therapy arm. The grey lines represent the observed values by each test person at day 1, 7 and 21. *, p < 0.05; *** p < 0.001; p-values were calculated using the Mann-Whitney test.

A total of 107 samples were collected from 19 test persons in the crossover design who inhaled either Tyloxapol or saline. The primary end point defined as sputum weight after three weeks of treatment showed a statistically significant difference (p = 0.041) between Tyloxapol and saline (Fig 3C). This was also true for the explorative comparison of the two treatment phases at day 1: p < 0.001 and after 1 week: p = 0.042 (Fig 3A and 3B).

In the explorative analysis at day 1, the measured sputum mean weight for the placebo arm was 2.39 g (95% CI 1.68–3.11 g) and for the Tyloxapol group, it was two times greater (5.07 g, 95% CI 3.46–6.67 g). At day 7, the exploratively measured values were 2.67 g (95% CI 1.51–3.84 g) and 3.88 g (95% CI 2.83–4.94 g) for the placebo group and the Tyloxapol group, respectively. At day 21, the measured values were 2.63 g (95% CI 1.73–3.53 g) for the placebo group and 4.03 g (95% CI 2.34–5.73 g) for the Tyloxapol group.

In addition an explorative longitudinal model was conducted on the basis of the weight of the expectorated sputum at days 1, +7 and +21. A significant treatment effect of Tyloxapol on sputum weight compared to the baseline treatment was found (p<0.001, Fig 3D). On average, the group treated with Tyloxapol expectorated 1.85 g more sputum compared to the placebo group at the different time points.

### Sputum weight in active smokers and ex-smokers

**Fig 4:**
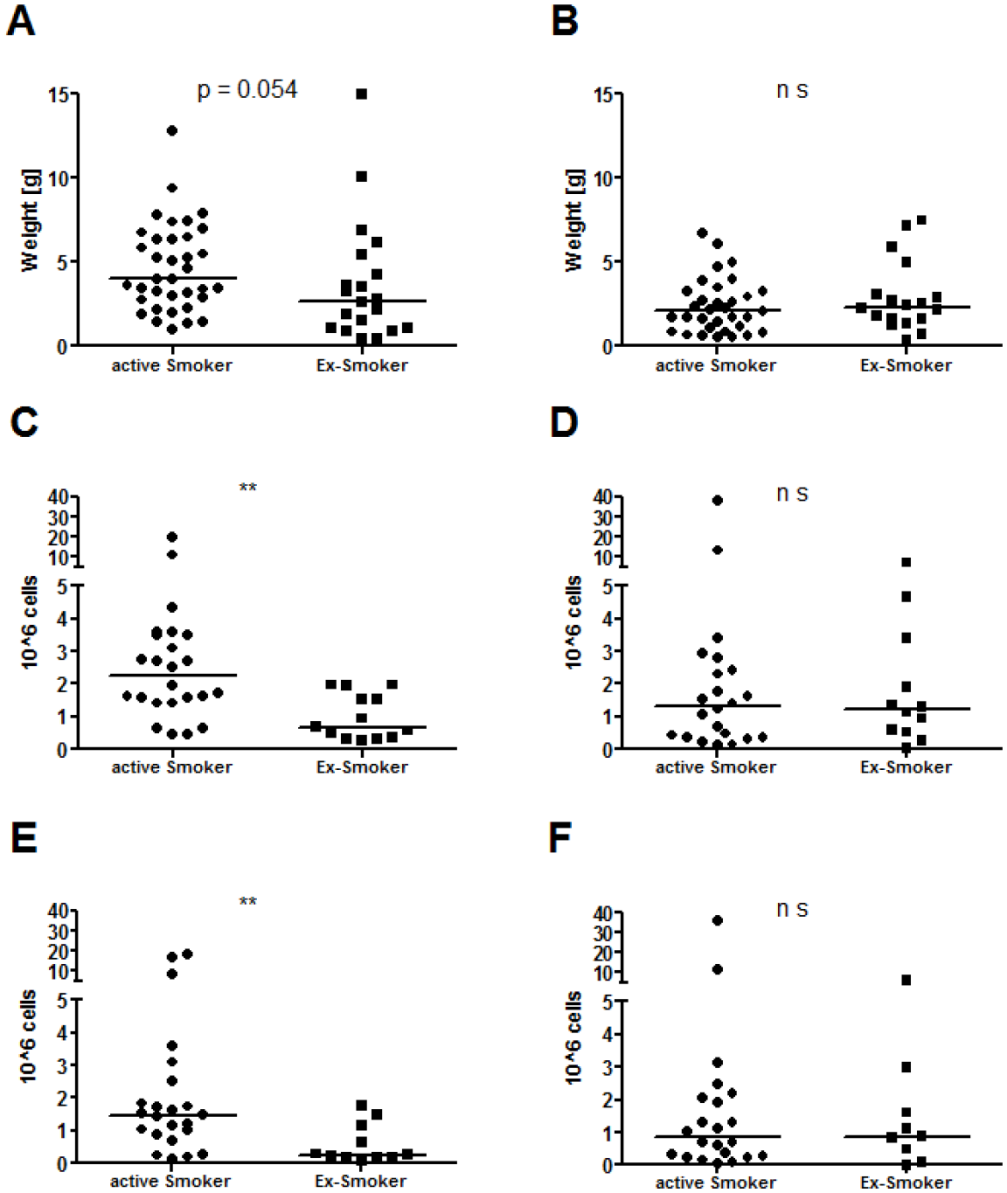
Sputum weight and cells comparing smokers and non-smokers during treatment with Tyloxapol and saline. (A, C and E) represent the Tyloxapol group, and (B, D and F) represent the saline group. (A-B) Sputum weight before processing. (C-D) the total cells and (E-F) the neutrophils. Sample size: A: n = 36 vs. 20, B: n = 33 vs. 18, C: n = 24 vs. 13, D: n = 22 vs. 12, E: n = 23 vs. 11, F: n = 22 vs. 9. Graphs show the pooled patients at all 3 visits and are separated by smoking status. Bars represent the median. ** p < 0.01; p-values were calculated using the Mann-Whitney test.

No difference was found in sputum weight at each visit and treatment phase for active smokers and ex-smokers. Even after pooling all the sputum samples of each treatment phase, no difference was found in the pre-selected sputum weight (primary parameter). However, we were able to exploratively detect a trend in the Tyloxapol group in which active smokers expectorated more sputum than ex-smokers (p = 0.054) (Fig 4A and 4B). Interestingly, the number of total cells (p = 0.002) and neutrophils (p = 0.006) were only higher in the group of pooled smokers during the Tyloxapol treatment (Fig 4C and 4E). At the same time, these parameters showed no evidence of differences during the placebo phase (Fig 4D and 4F).

### Sputum Cells

Sputum cells are shown in Table 2. Interestingly, there was a median increase in total cells during the saline treatment from 1.09 (range: 0.02–13.29) (day 1) to 1.38 (0.13–38.25) x 10^6^ cells (week 3). In contrast, during the Tyloxapol treatment, total cells decreased from 1.64 (0.32–19.72) (day 1) to 1.18 (0.22–19.77) x 10^6^ cells (week 3). In particular, neutrophils decreased from 1.32 (0.07–16.86) to 0.86 (0.17–18.53) x 10^6^ cells in that time period, indicating that Tyloxapol might have some anti-neutrophilic properties. In addition, macrophages showed a similar trend under the saline treatment (day 1: 0.16 to 0.32 x 10^6^ cells after three weeks), whereas under the Tyloxapol treatment, macrophages decreased (0.47 at day 1 to 0.19 x 10^6^ cells at 3 weeks). In addition to these trends, which were not significant, we were able to detect significantly more macrophages on the first day of treatment with Tyloxapol compared with the placebo (p = 0.010).

**Table 2:** Cells and mediators in sputum of patients per protocol. Values are the median and range; whereas the cell differentiation for macrophages, neutrophils and lymphocytes are the median and range *10^6^.

	Day 1 of treatment		3 weeks of treatment	
	Saline	Tyloxapol	Saline	Tyloxapol
Total Cells	**1.09** (0.02–13.29)	**1.64** (0.32–19.72)	**1.38** (0.13–38.25)	**1.18** (0.22–19.77)
Percentage of living cells	**68.57** (26.67–100)	**73.33** (29.16–95.92)	**63.04** (7.44–95.84)	**63.08** (21.74–100)
Macrophages x 10^4^/ml	**0.16** (0.01–0.75)**	**0.47** (0.06–2.68)**	**0.32** (0.01–3.92)	**0.19** (0.03–1.76)
Macrophages (%)	**17.10** (2.00–54.90)	**32.91** (6.50–82.75)	**27.24** (2.50–84.00)	**23.80** (2.25–69.75)
Neutrophils x 10^4^/ml	**0.83** (0.01–11.83)	**1.32** (0.07–16.86)	**0.95** (0.03–36.05)	**0.86** (0.17–18.53)
Neutrophils (%)	**76.84** (33.33–89.00)	**63.14** (12.50–91.50)	**67.71** (10.50–94.25)	**70.68** (27.00–97.00)
Lymphocytes x 10^4^/ml	**0.02** (0.00–1.20)	**0.04** (0.00–1.33)	**0.04** (0.00–1.24)	**0.03** (0.00–0.19)
Lymphocytes (%)	**5.41** (0.25–29.75)	**3.12** (0.00–9.25)	**4.42** (0.75–24.00)	**4.98** (0.25–41.00)
Eosinophils x 10^4^/ml	**0.01** (0.00–0.11)	**0.01** (0.00–0.08)	**0.01** (0.00–0.07)	**0.01** (0.00–0.03)
Eosinophils (%)	**0.65** (0.00–1.96)	**0.75** (0.00–2.50)	**0.50** (0.00–2.25)	**0.49** (0.00–0.75)
IL-1β (pg/ml)	**419** (31–31921)	**486** (82–17897)	**584** (35–46538)	**404** (23–16135)
IL-6 (pg/ml)	**1735** (23–5757)	**1586** (83–6684)	**2111** (17–10764)	**1400** (189–13692)
IL-8 (pg/ml)	**32898** (3306–81328)	**48531** (3811–141065)	**45767** (3240–339898)	**31940** (4305–209158)
MMP-9 (ng/ml)	**148** (0–258)	**197** (0–247)	**204** (0–260)	**191** (16–275)
LTB4 (pg/ml)	**2477** (481–10075)	**4643** (812–30000)	**2060** (727–24832)	**4111** (895–30000)
Elastase (ng/ml)	**40** (0–59)	**44** (16–53)	**41** (0–55)	**42** (0–59)

** p < 0.01 for saline vs. Tyloxapol; p-values were calculated using the t-test.

### Cytokines *in vivo*

Inflammatory biomarkers (IL-1β, IL-6, and IL-8) in the sputum are shown in Table 2. Interestingly, there was an increase in median levels of all inflammatory cytokines during the saline treatment from day 1 to week 3, whereas during the Tyloxapol treatment, cytokines remained unchanged. However, due to the small sample size and the large individual variation in sputum cytokines, these differences were not significant.

### Macrophage activation test *in vitro*

**Fig 5:**
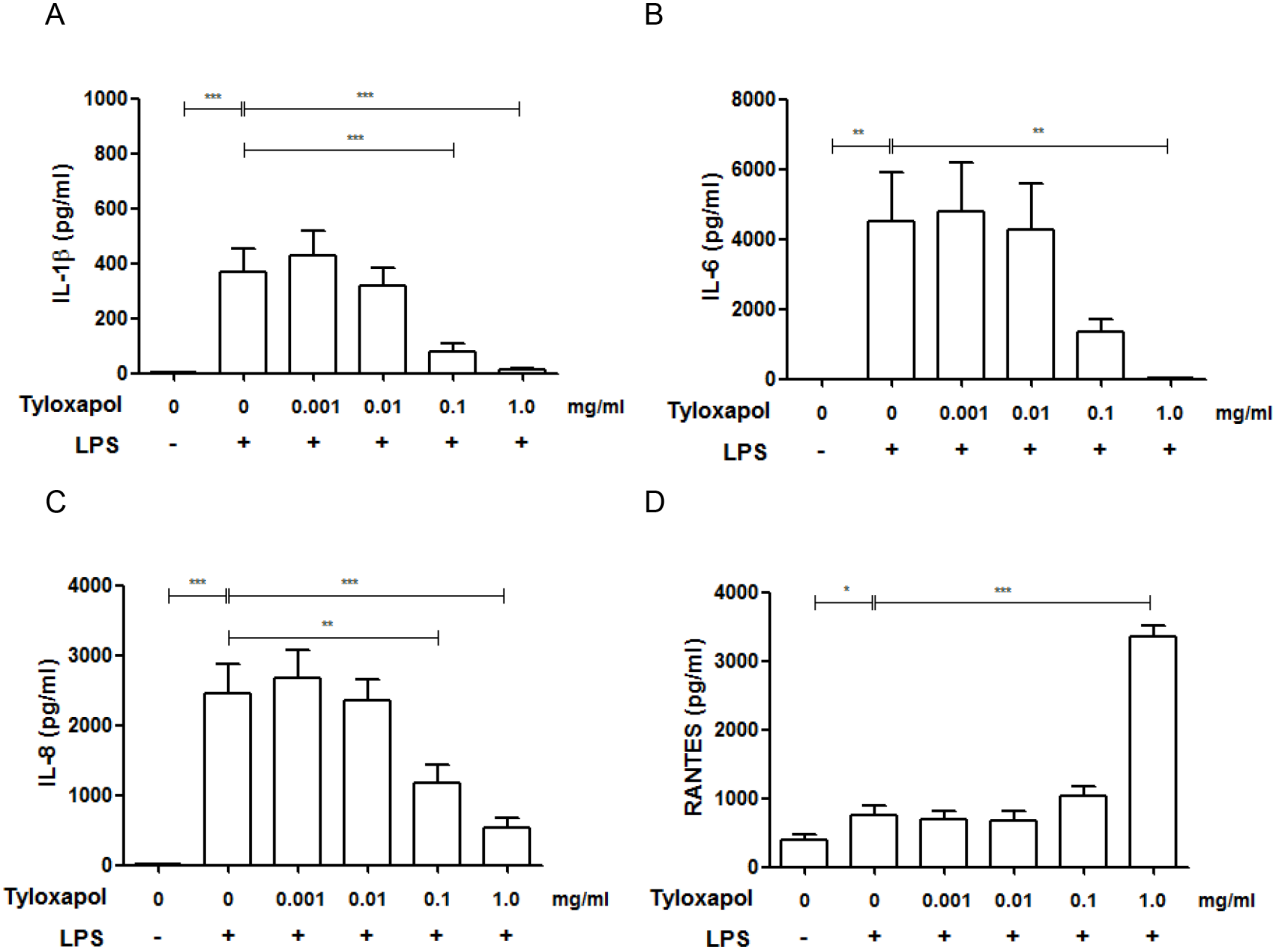
Cytokine release after macrophage activation test. (A-D) Cytokine release, after 24-hr LPS-stimulation of whole blood cells incubated with different concentrations of Tyloxapol in *vitro* as measured by the CBA. * p < 0.05; ** p < 0.01; *** p < 0.001.

The Macrophage activation test revealed distinct anti-inflammatory effects of Tyloxapol at concentrations of 0.1 and 1.0 mg/ml *in vitro*. Macrophages released significantly less IL-1β, IL-6 and IL-8 compared with LPS-stimulation only (p < 0.001, p < 0.01 and p < 0.001), as shown in Fig 5, whereas RANTES was significantly increased at 1.0 mg/ml Tyloxapol (p<0.001).

### Cell viability

**Fig 6:**
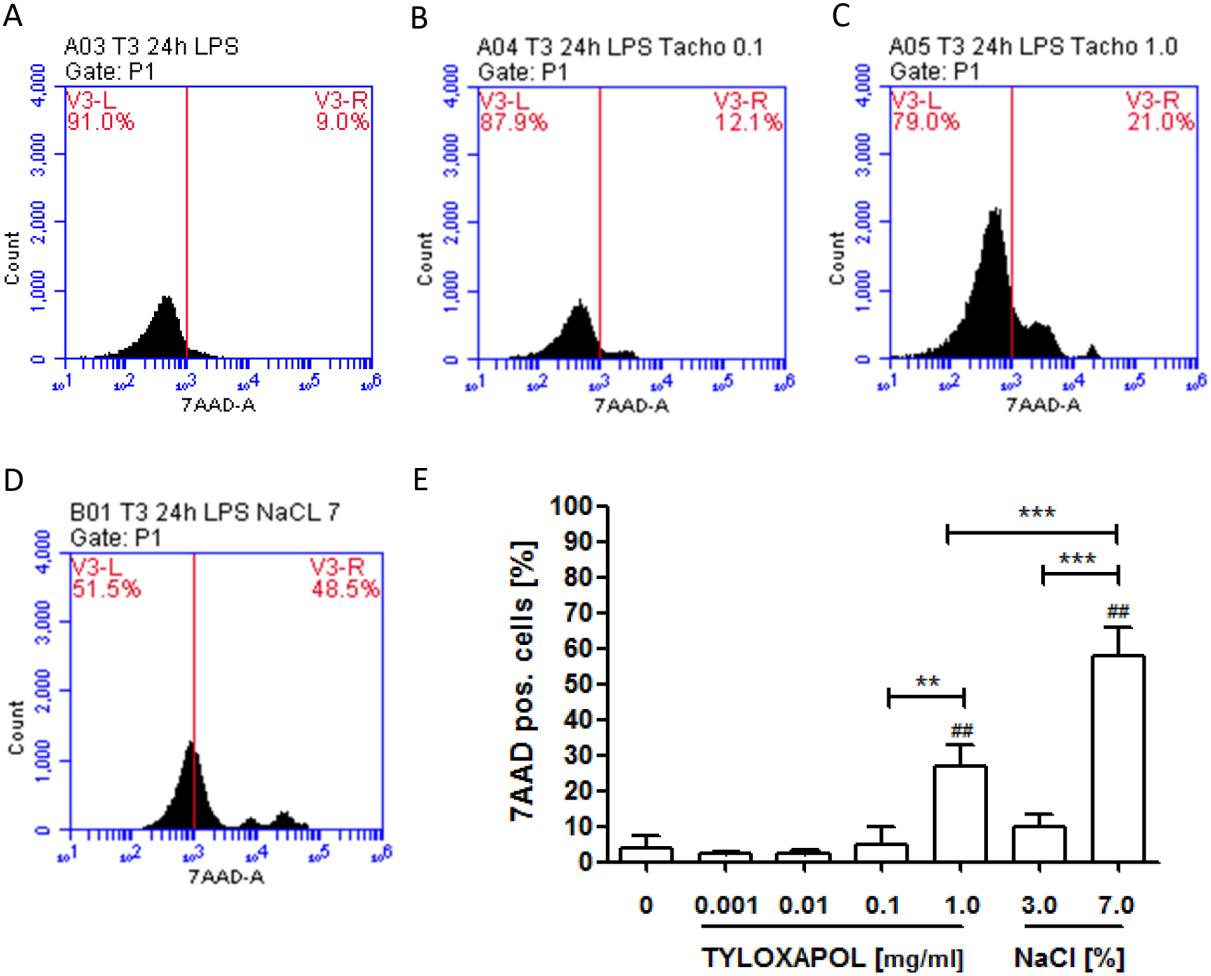
Effect of Tyloxapol on cell viability. Cell viability of LPS treated whole blood cells in the presence of different concentrations of Tyloxapol (0, 0.001, 0.01, 0.1, 1.0 mg/ml) compared with hypertonic saline (3.0, 7.0%) after 24 hours was measured by 7AAD-staining using flow cytometry. (A-D) Histograms of flow cytometric analysis. (E) Quantitative analysis of four independent analysis. ** p < 0.01; *** p < 0.001, ## p<0.01 compared to Tyloxapol 0 mg/ml.

Tyloxapol did not affect cell viability up to a concentration of 0.1 mg/ml (Fig 6). At a concentration of 1 mg/ml Tyloxapol, the rate of dead cells raised up to 28.74% but killing was significantly lower than with hypertonic saline after 24 hours (w/o Tyloxapol: 1.86% (95% CI 0.80–2.92%); Tyloxapol 1 mg/ml: 28.74% (95% CI 15.18–42.30%), p < 0.01; hypertonic saline: 60.33% (95% CI 43.33–78.10%), p < 0.001).

### ELISA

No difference in concentrations of Elastase, MMP-9 and LTB4 was found between the Tyloxapol and saline treated groups (Table 2).

### Lung function analysis

Lung function parameters (FVC, FEV_1_, MEF25, RV, and RV/TLC) were measured before and after reversibility testing and 30 and 60 minutes after inhalation. The analysis included day 1, week 1 and week 3 data. As expected, there was a small increase in FEV_1_ after reversibility testing (see Table 1). However, there were no significant differences at 30 and 60 minutes (data not shown) and between the treatment arms at day 1, day 7 and after 3 weeks for all parameters measured (see Table 3).

**Table 3:** Lung function of Patients per protocol. Values are the mean and standard error of mean.

	Day 1 of treatment	1 Week of treatment	3 Weeks of treatment
**Saline**			
FVC (%)	**77.26** (2.90)	**77.21** (2.85)	**73.47** (2.96)
Pre-FEV_1_ (%)	**55.26** (3.70)	**54.00** (2.93)	**53.37** (3.87)
Post-FEV_1_ (%)	**58.26** (2.78)	**59.89** (3.09)	**60.37** (3.56)
Pre-FEV_1_/FVC	**55.85** (2.91)	**54.73** (2.54)	**54.15** (2.82)
Post-FEV_1_/FVC	**56.16** (2.85)	**57.09** (2.68)	**56.10** (3.04)
MEF 25 (%)	**24.68** (2.61)	**23.58** (2.13)	**25.16** (2.96)
RV (%)	**183.32** (11.81)	**201.32** (20.08)	**186.42** (16.29)
RV/TLC (%)	**150.11** (4.59)	**152.42** (5.78)	**149.11** (5.88)
**Tyloxapol**			
FVC (%)	**75.74** (3.33)	**77.37** (2.85)	**74.84** (3.61)
Pre-FEV_1_ (%)	**53.05** (3.11)	**54.16** (3.37)	**52.00** (3.06)
Post-FEV_1_ (%)	**61.00** (3.20)	**61.47** (3.45)	**59.00** (2.90)
Pre-FEV_1_/FVC	**54.45** (2.55)	**54.23** (2.46)	**51.74** (3.05)
Post-FEV_1_/FVC	**57.55** (2.56)	**57.30** (2.85)	**57.08** (3.19)
MEF 25 (%)	**22.37** (1.55)	**23.84** (2.33)	**25.11** (2.99)
RV (%)	**176.05** (14.42)	**191.74** (22.26)	**200.89** (27.83)
RV/TLC (%)	**146.11** (5.72)	**147.11** (6.19)	**148.63** (5.84)

### Symptom scores

The CAT was used to measure the symptom load in COPD. The mean score before treatment with saline (20.79, SEM: 1.52 points) or Tyloxapol (20.21, SEM: 1.12 points) was similar and did not change during treatment (Table 4).

**Table 4:** Symptom scores, quality of life and Likert score of patients per protocol. Values are the mean and standard error of mean.

	Day 1 of treatment	1 week of treatment	3 weeks of treatment
**Saline**			
CAT (points)	**20.79** (1.52)	**18.74** (1.14)	**19.37** (1.25)
SGRQ (%)	**51.38** (3.12)		**47.36** (3.42)
BDI/TDI (points)	**7.71** (0.65)	**0.94** (0.47)	**1.18** (0.47)
Likert 1 (points)	**2.63** (0.34)	**2.95** (0.35)	**2.95** (0.31)
Likert 2 (points)	**2.63** (0.39)	**3.00** (0.28)	**3.21** (0.33)
**Tyloxapol**			
CAT (points)	**20.21** (1.12)	**18.37** (1.28)	**18.32** (1.26)
SGRQ (%)	**53.15** (3.70)		**49.65** (3.57)
BDI/TDI (points)	**7.76** (0.67)	**0.65** (0.70)	**1.47** (0.80)
Likert 1 (points)	**3.05** (0.44)	**3.05** (0.34)	**3.21** (0.39)
Likert 2 (points)	**3.53** (0.42)	**3.16** (0.34)	**3.47** (0.33)

Dyspnoea and quality of life were assessed by the BDI, the TDI and the SGRQ. As shown in Table 4, there were no significant changes during either treatment period.

In addition, we analysed the ease of sputum production by an analogue scale during the study modified to Kellett et al [27]. There was no significant difference in the mucous congestion (Likert 1) and ease of expectoration (Likert 2) visual analogue scores between the different treatment phases. However, multiple comparisons between treatment visits show that median values for Likert 1 and Likert 2 at every time point were greater with Tyloxapol. Again, most likely due to the small number of patients, these differences were not significant.

### Safety report

During the three weeks of treatment, patients performed more than 1000 inhalations either with saline or with Tyloxapol. Despite the high number of inhalations, only a few AEs (n = 27) were recorded in the treated population, indicating that inhalations of saline and Tyloxapol are very safe procedures (see Table 5). However, 20 of these AEs were recorded during the Tyloxapol treatment (in eleven patients of the treated population) compared to seven during saline (in six of the treated population), showing that Tyloxapol has a higher risk of side effects than saline. It is well known that after Tyloxapol inhalation, the first deep breaths can be followed by an initial urge to cough, which may disappear after moistening the mucous membrane. As expected, there was a suggestion of more patients experiencing shortness of breath or coughing after Tyloxapol vs. saline (n = 10 vs. n = 3). The estimated odds ratio of relevant adverse was 3.49 (95% CI 0.81–19.51; p = 0.16). However, it is important to note that all reported AEs related to the study were non-serious and resolved without treatment. Thus, no events had to be reported according to the medical product law in Germany.

**Table 5:** Number of AEs at treatment with Tyloxapol vs. saline of the treated population (n = 21). All AEs were mild in nature and self limited and possibly related to treatment except one patient* who discontinued the study due to intestinal bleeding.

		Tyloxapol	Saline
Shortness of breath		5	1
Coughing		5	2
Cold		3	0
Headache		2	0
Mucositis		0	2
Exacerbation		0	1
Other		5	1*
**Total**		**20**	**7**
	Patients with AEs	**11**	**6**
**Relevant AEs**	Including shortness of breath and Coughing	**10**	**3**
	Patients with relevant AEs	**8**	**3**

## Discussion

In the classical chronic bronchitis phenotype of COPD, mucus hypersecretion is the key presenting symptom, and its contribution to airflow obstruction is well established. Mucus hypersecretion is a consequence of noxious gas exposure [28], acute viral or bacterial infection, and/or ongoing inflammatory cell and mucin gene activation. Drugs to promote mucus clearance may reduce the sequelae of chronic bronchitis and COPD as Cerveri et al. state [29]. However, the regular use of mucolytics in COPD has controversial results [30–32]. Although a few patients with viscous sputum may benefit from mucolytics, the recent GOLD report 2015 did not recommend the widespread use of these agents [10, 33]. Thus, there is an unmet need for potent mucolytics that promote sputum expectoration and may have new anti-inflammatory properties.

The aim of the present study was to assess the effects of the mucolytic agent, Tyloxapol, which has potent secretolytic action and may down regulate inflammation. As reported by Paez, we were able to demonstrate that sputum expectoration was significantly better with Tyloxapol than with saline, as measured by sputum weight [17]. But our data is difficult to compare to Paez. In the former study patients inhaled 35ml with 0.125% Tyloxapol once a day for 5 days a week. Our patients inhaled 3 times a day 5 ml with 1% Tyloxapol for 7 days a week, which is more suitable for daily practice. Additionally Paez calculated sputum volume and dry weight per hour while we calculate total and selected sputum plaques after inhalation.

On average in our study, COPD patients treated with Tyloxapol expectorated 1.85 g more sputum compared to saline at all time points investigated. The expectorated sputum was in the range of hypertonic saline [34]. Some studies have shown a positive effect of mannitol [35]. In contrast, many mucolytic agents such as N-acetylcysteine [36], DNase and isotonic saline [37] have failed to show an effect on sputum weight, and have had little effect on changes in sputum volume or viscosity [38].

Next, we addressed the question of whether smoking status was a possible confounder [39, 40]. No differences were found for any of the visits and treatment phases. This lack of positive finding was most likely related to the small number of patients (active smokers (n = 12) and ex-smokers (n = 7)). To overcome this limitation, the sputum samples were pooled at each treatment phase. Again, no differences were found for the Tyloxapol or saline treatments, but we were able to detect a trend in the Tyloxapol group in which active smokers expectorated more sputum than ex-smokers. Interestingly, the number of total cells (p = 0.002) and neutrophils (p = 0.006) were only higher in the group of pooled smokers during the Tyloxapol treatment. At the same time, these parameters showed no evidence of differences during the placebo phase. This finding is difficult to explain but may be explained by different sputum viscosities and a high potential to resolve plaques in the lung when active smokers inhale Tyloxapol.

Lung function parameters remained stable during the study, and no treatment effect was shown. There are conflicting reports about the effects of mucolytics on lung function in various respiratory diseases such as cystic fibrosis, bronchiectasis and COPD. Generally, the lung function changes have been small, or the authors have failed to show any benefit. For hypertonic saline, different results have been published. Two studies with 28 and 12 patients only showed an increase in FEV_1_ [27, 41], whereas another with 10 patients detected no change [42]. For N-Acetylcysteine (24 vs. 27; 186 vs. 168 and 19 vs. 19 patients) [36, 43–45] and mannitol (9 and 12 patients) [46, 47], stable or even slightly decreasing lung function has been described. For DNase I, we found two studies stating an increase (20 vs. 21 and 156 vs. 176 patients) [48, 49], and one a decrease with 172 vs. 176 patients [50] in lung function in cystic fibrosis. Only a very few studies have been performed on COPD.

Mucous congestion and ease of expectoration (Likert 1 and 2) were unchanged between the different treatment phases. However, multiple comparisons between treatment visits show that median values of mucous congestion and ease of expectoration at every time point were greater with Tyloxapol. Again, most likely due to the small number of patients, these differences were not significant. In addition, our subjective measure of sputum expectoration deserves comment. Because there were no pre-existing subjective measures, we constructed our own Likert scale for a subjective assessment of mucous congestion and ease of expectoration; as a result, its reliability and validity can be questioned.

In contrast to other studies with mucolytics, we did not detect any change in our quality of life scores [27, 46, 51]. This is difficult to explain but may be in part related to the relatively short treatment period. Additionally, other mucolytics have reportedly been ineffective as well [9, 35, 36, 52].

Alternatively, it is well known that saline given through an efficient nebulizer system enables patients to expectorate sputum [53, 54]. And it is broadly used in clinical practice as such. Therefore, the question of whether saline is an appropriate placebo has to be discussed. Recently, it was shown that nebulized saline can be used as a placebo in lung function studies, but it cannot be used as a placebo in trials assessing symptom relief [55]. Thus, placebo effects can be clinically meaningful solely due to the patient’s expectation on the patient-reported outcome or to reporting bias (e.g., the wish to please the investigator).

In addition during the three weeks of treatment all patients together performed more than 1000 inhalations either with saline and with Tyloxapol. Despite the high number of inhalations, only a few AEs (n = 27) were recorded, indicating that inhalations of saline and Tyloxapol are very safe procedures. All AEs were non-serious, self-limiting and resolved without treatment.

Not only was sputum expectoration better after Tyloxapol inhalation, but interestingly, a number of secondary parameters (total cells, neutrophils and cytokines) also improved with Tyloxapol, although most of these improvements failed to be significant due to the small sample size studied. The question of whether the small decrease in inflammation was solely due to expectoration of purulent sputum and was merely incidental or an active property of Tyloxapol has to be investigated.

To overcome these study limitations, we used the macrophage activation test in *vitro* to study the anti-inflammatory effect of Tyloxapol. We were able to show distinct anti-inflammatory effects of Tyloxapol at concentrations of 0.1 and 1.0 mg/ml *in vitro*. Macrophages released significantly less IL-1β, IL-6 and IL-8 compared with LPS-stimulation only, as shown in Fig 5, whereas RANTES was significantly increased, indicating an active effect of Tyloxapol. Decrease of inflammation was not due to cell death indicated by the cell viability testing. Our data are in good agreement with the findings of GHIO et al [20]. These authors characterized the anti-inflammatory potency of Tyloxapol and showed that Tyloxapol inhibited activation of the transcription factor nuclear factor-kappa-B and reduced several inflammatory proteins, such as TNF-α, IL-1β, IL-6 and IL-8, in cell culture. These anti-inflammatory effect have been described for hypertonic saline, acetylcysteine and Tyloxapol and might be even more useful than secretolytic effects in the long run for COPD patients, especially in the exacerbating/inflammatory phenotype of COPD. Indeed, several clinical studies with expectorants did show a clinical effect in reduction of exacerbations. [43, 44, 56, 57]

The study has several limitations. As a proof of concept study the treatment period of Tyloxapol was too short to see any effects on quality of life and exacerbations. The significant anti-inflammatory effect of Tyloxapol was shown in *vitro* only, and the question of whether this effect can be shown in *vivo* in a larger number of patients remains unanswered.

In conclusion, our study demonstrated that inhalation of Tyloxapol in patients with COPD is safe and superior to saline and shows some anti-inflammatory effects.

## Supporting Information

S1 CONSORT Checklist (DOC)

S1 Dataset Minimal data set. (XLSX)

S1 Study Protocol (PDF)

## References

[1] Vestbo J, Hogg JC. Convergence of the epidemiology and pathology of COPD. Thorax 2006; 61(1):86–8. 16227325 10.1136/thx.2005.046227 PMC2080699

[2] Fletcher C, Peto R. The natural history of chronic airflow obstruction. British medical journal 1977; 1(6077):1645–8. 871704 10.1136/bmj.1.6077.1645 PMC1607732

[3] Vestbo J, Prescott E, Lange P. Association of chronic mucus hypersecretion with FEV1 decline and chronic obstructive pulmonary disease morbidity. Copenhagen City Heart Study Group. American journal of respiratory and critical care medicine 1996; 153(5):1530–5. 8630597 10.1164/ajrccm.153.5.8630597

[4] de Marco R, Accordini S, Cerveri I, Corsico A, Antó JM, Künzli N et al Incidence of chronic obstructive pulmonary disease in a cohort of young adults according to the presence of chronic cough and phlegm. American journal of respiratory and critical care medicine 2007; 175(1):32–9. 17008642 10.1164/rccm.200603-381OC

[5] BRUMFITT W, WILLOUGHBY ML, BROMLEY LL. An evaluation of sputum examination in chronic bronchitis. Lancet (London, England) 1957; 273(7009):1306–9. 10.1016/s0140-6736(57)91637-9 13492632

[6] Kohansal R, Martinez-Camblor P, Agustí A, Buist AS, Mannino DM, Soriano JB. The natural history of chronic airflow obstruction revisited: an analysis of the Framingham offspring cohort. American journal of respiratory and critical care medicine 2009; 180(1):3–10. 10.1164/rccm.200901-0047OC 19342411

[7] Decramer M, Janssens W. Mucoactive therapy in COPD. European respiratory review: an official journal of the European Respiratory Society 2010; 19(116):134–40. 20956182 10.1183/09059180.00003610 PMC9682579

[8] Balsamo R, Lanata L, Egan CG. Mucoactive drugs. European respiratory review: an official journal of the European Respiratory Society 2010; 19(116):127–33. 20956181 10.1183/09059180.00003510 PMC9682576

[9] Poole P, Chong J, Cates CJ. Mucolytic agents versus placebo for chronic bronchitis or chronic obstructive pulmonary disease. The Cochrane database of systematic reviews 2015; 7:CD001287 10.1002/14651858.CD001287.pub5 26222376

[10] Marc Decramer MD. Global Strategy for Diagnosis, Management, and Prevention of COPD (Updated 2015) [cited 2015 Aug 21]. Available from: URL:http://www.goldcopd.org/guidelines-global-strategy-for-diagnosis-management.html.

[11] HUTSCHENREUTER K. Die Bedeutung moderner Netzmittel für die Chirurgie. Der Chirurg; Zeitschrift für alle Gebiete der operativen Medizen 1958; 29(6):252–8. 13547306

[12] WAGENEDER FM. [Tacholiquin in the pre- and postoperative phase of thoracic surgical interventions]. Münchener medizinische Wochenschrift (1950) 1962; 104:2296–8. 13998187

[13] PALMER KN. A new mucolytic agent by aerosol for inhalation in chronic bronchitis. Lancet (London, England) 1961; 2(7206):802–4. 10.1016/s0140-6736(61)91090-x 14483415

[14] RAVENEL SF. New technique of humidification in pediatrics. Journal of the American Medical Association 1953; 151(9):707–11. 13011077

[15] CONLEY JJ. Diagnosis and treatment of encrustations in the trachea: their relation to radical surgery of the head and neck. Journal of the American Medical Association 1954; 154(10):829–32. 13129048 10.1001/jama.1954.02940440027008

[16] TAINTER ML, NACHOD FC, BIRD JG. Alevaire as a mycolytic agent. The New England journal of medicine 1955; 253(18):764–7. 13266038 10.1056/NEJM195511032531804

[17] Paez PN, Miller WF. Surface Active Agents in Sputum Evacuation: A Blind Comparison with Normal Saline Solution and Distilled Water. Chest 1971; 60(4). 10.1378/chest.60.4.312 4940230

[18] Wilde A. Oberflächenspannungs- und Viskositäts-Messungen an Sputa unter dem Einfluss von Tacholiquin. Notabene Medici 1978; (8. Heft 1.).

[19] FARBER SM, BENIOFF MA, SMITH JD. Planned care for patients with bronchiectasis. California medicine 1955; 82(2):102–6. 13230925 PMC1532464

[20] Ghio AJ, Marshall BC, Diaz JL, Hasegawa T, Samuelson W, Povia D et al Tyloxapol inhibits NF-kappa B and cytokine release, scavenges HOCI, and reduces viscosity of cystic fibrosis sputum. American journal of respiratory and critical care medicine 1996; 154(3 Pt 1):783–8. 8810619 10.1164/ajrccm.154.3.8810619

[21] Criée CP, Sorichter S, Smith HJ, Kardos P, Merget R, Heise D et al Body plethysmography—its principles and clinical use. Respiratory Medicine 2011; 105(7):959–71. 10.1016/j.rmed.2011.02.006 21356587

[22] Schulze J, Voss S, Zissler U, Rose MA, Zielen S, Schubert R. Airway responses and inflammation in subjects with asthma after four days of repeated high-single-dose allergen challenge. Respiratory research 2012; 13:78 22989372 10.1186/1465-9921-13-78 PMC3445853

[23] Woolhouse IS, Bayley DL, Stockley RA. Effect of sputum processing with dithiothreitol on the detection of inflammatory mediators in chronic bronchitis and bronchiectasis. Thorax 2002; 57(8):667–71. 12149524 10.1136/thorax.57.8.667 PMC1746392

[24] Rosewich M, Zissler UM, Kheiri T, Voss S, Eickmeier O, Schulze J et al Airway inflammation in children and adolescents with bronchiolitis obliterans. Cytokine 2015; 73(1):156–62. 10.1016/j.cyto.2014.10.026 25748838

[25] Schmitt-Grohé S, Hippe V, Igel M, von Bergmann K, Posselt HG, Krahl A et al Lipopolysaccharide binding protein, cytokine production in whole blood, and lipoproteins in cystic fibrosis. Pediatric research 2005; 58(5):903–7. 16183806 10.1203/01.PDR.0000182598.98167.24

[26] Eickmeier O, Huebner M, Herrmann E, Zissler U, Rosewich M, Baer PC et al Sputum biomarker profiles in cystic fibrosis (CF) and chronic obstructive pulmonary disease (COPD) and association between pulmonary function. Cytokine 2010; 50(2):152–7. 10.1016/j.cyto.2010.02.004 20181491

[27] Kellett F, Robert NM. Nebulised 7% hypertonic saline improves lung function and quality of life in bronchiectasis. Respiratory Medicine 2011; 105(12):1831–5. 10.1016/j.rmed.2011.07.019 22018993

[28] Dijkstra AE, de Jong K, Boezen HM, Kromhout H, Vermeulen R, Groen HJM et al Risk factors for chronic mucus hypersecretion in individuals with and without COPD: influence of smoking and job exposure on CMH. Occupational and environmental medicine 2014; 71(5):346–52. 10.1136/oemed-2013-101654 24642640

[29] Cerveri I, Brusasco V. Revisited role for mucus hypersecretion in the pathogenesis of COPD. European respiratory review: an official journal of the European Respiratory Society 2010; 19(116):109–12. 20956178 10.1183/09059180.00002710 PMC9682580

[30] Allegra L, Cordaro CI, Grassi C. Prevention of acute exacerbations of chronic obstructive bronchitis with carbocysteine lysine salt monohydrate: a multicenter, double-blind, placebo-controlled trial. Respiration; international review of thoracic diseases 1996; 63(3):174–80. 8739489 10.1159/000196540

[31] Guyatt GH, Townsend M, Kazim F, Newhouse MT. A controlled trial of ambroxol in chronic bronchitis. Chest 1987; 92(4):618–20. 3308343 10.1378/chest.92.4.618

[32] Petty TL. The National Mucolytic Study. Results of a randomized, double-blind, placebo-controlled study of iodinated glycerol in chronic obstructive bronchitis. Chest 1990; 97(1):75–83. 2403903 10.1378/chest.97.1.75

[33] Celli BR, MacNee W. Standards for the diagnosis and treatment of patients with COPD: a summary of the ATS/ERS position paper. The European respiratory journal 2004; 23(6):932–46. 15219010 10.1183/09031936.04.00014304

[34] Kellett F, Redfern J, McL Niven R. Evaluation of nebulised hypertonic saline (7%) as an adjunct to physiotherapy in patients with stable bronchiectasis. Respiratory Medicine 2005; 99(1):27–31. 15672845 10.1016/j.rmed.2004.05.006

[35] Bilton D, Daviskas E, Anderson SD, Kolbe J, King G, Stirling RG et al Phase 3 randomized study of the efficacy and safety of inhaled dry powder mannitol for the symptomatic treatment of non-cystic fibrosis bronchiectasis. Chest 2013; 144(1):215–25. 10.1378/chest.12-1763 23429964

[36] Dueholm M, Nielsen C, Thorshauge H, Evald T, Hansen NC, Madsen HD et al N-acetylcysteine by metered dose inhaler in the treatment of chronic bronchitis: a multi-centre study. Respiratory Medicine 1992; 86(2):89–92. 1615189 10.1016/s0954-6111(06)80220-9

[37] Gallon AM. Evaluation of nebulised acetylcysteine and normal saline in the treatment of sputum retention following thoracotomy. Thorax 1996; 51(4):429–32. 8733499 10.1136/thx.51.4.429 PMC1090682

[38] Sathe NA, Krishnaswami S, Andrews J, Ficzere C, McPheeters ML. Pharmacologic Agents That Promote Airway Clearance in Hospitalized Subjects: A Systematic Review. Respiratory care 2015; 60(7):1061–70. 10.4187/respcare.04086 25944943

[39] Faner R, Gonzalez N, Cruz T, Kalko SG, Agustí A. Systemic inflammatory response to smoking in chronic obstructive pulmonary disease: evidence of a gender effect. PloS one 2014; 9(5):e97491 10.1371/journal.pone.0097491 24830457 PMC4022517

[40] Hogg JC, Chu F, Utokaparch S, Woods R, Elliott WM, Buzatu L et al The nature of small-airway obstruction in chronic obstructive pulmonary disease. The New England journal of medicine 2004; 350(26):2645–53. 15215480 10.1056/NEJMoa032158

[41] Donaldson SH, Bennett WD, Zeman KL, Knowles MR, Tarran R, Boucher RC. Mucus clearance and lung function in cystic fibrosis with hypertonic saline. The New England journal of medicine 2006; 354(3):241–50. 16421365 10.1056/NEJMoa043891

[42] Robinson M, Hemming AL, Regnis JA, Wong AG, Bailey DL, Bautovich GJ et al Effect of increasing doses of hypertonic saline on mucociliary clearance in patients with cystic fibrosis. Thorax 1997; 52(10):900–3. 9404379 10.1136/thx.52.10.900 PMC1758438

[43] Poole PJ, Black PN. Oral mucolytic drugs for exacerbations of chronic obstructive pulmonary disease: systematic review. BMJ (Clinical research ed.) 2001; 322(7297):1271–4. 10.1136/bmj.322.7297.1271 PMC31920 11375228

[44] Decramer M, Rutten-van Mölken M, Dekhuijzen PR, Troosters T, van Herwaarden C, Pellegrino R et al Effects of N-acetylcysteine on outcomes in chronic obstructive pulmonary disease (Bronchitis Randomized on NAC Cost-Utility Study, BRONCUS): A randomised placebo-controlled trial. The Lancet 2005; 365(9470):1552–60. 10.1016/S0140-6736(05)66456-2 15866309

[45] Ayfer Aytemur Z, Baysak A, Ozdemir O, Köse T, Sayiner A. N-acetylcysteine in patients with COPD exacerbations associated with increased sputum. Wiener klinische Wochenschrift 2015; 127(7–8):256–61. 10.1007/s00508-014-0692-4 25595117

[46] Daviskas E, Anderson SD, Gomes K, Briffa P, Cochrane B, Chan H et al Inhaled mannitol for the treatment of mucociliary dysfunction in patients with bronchiectasis: effect on lung function, health status and sputum. Respirology (Carlton, Vic.) 2005; 10(1):46–56. 10.1111/j.1440-1843.2005.00659.x 15691238

[47] Robinson M, Daviskas E, Eberl S, Baker J, Chan HK, Anderson SD et al The effect of inhaled mannitol on bronchial mucus clearance in cystic fibrosis patients: a pilot study. The European respiratory journal 1999; 14(3):678–85. 10543292 10.1034/j.1399-3003.1999.14c30.x

[48] Shah PL, Scott SF, Knight RA, Marriott C, Ranasinha C, Hodson ME. In vivo effects of recombinant human DNase I on sputum in patients with cystic fibrosis. Thorax 1996; 51(2):119–25. 8711640 10.1136/thx.51.2.119 PMC473012

[49] McCoy K, Hamilton S, Johnson C. Effects of 12-week administration of dornase alfa in patients with advanced cystic fibrosis lung disease. Pulmozyme Study Group. Chest 1996; 110(4):889–95. 10.1378/chest.110.4.889 8874241

[50] O'Donnell AE, Barker AF, Ilowite JS, Fick RB. Treatment of idiopathic bronchiectasis with aerosolized recombinant human DNase I. rhDNase Study Group. Chest 1998; 113(5):1329–34. 9596315 10.1378/chest.113.5.1329

[51] Nicolson CHH, Stirling RG, Borg BM, Button BM, Wilson JW, Holland AE. The long term effect of inhaled hypertonic saline 6% in non-cystic fibrosis bronchiectasis. Respiratory Medicine 2012; 106(5):661–7. 10.1016/j.rmed.2011.12.021 22349069

[52] Tse HN, Raiteri L, Wong KY, Yee KS, Ng LY, Wai KY et al High-dose N-acetylcysteine in stable COPD: the 1-year, double-blind, randomized, placebo-controlled HIACE study. Chest 2013; 144(1):106–18. 10.1378/chest.12-2357 23348146

[53] Sutton PP, Gemmell HG, Innes N, Davidson J, Smith FW, Legge JS et al Use of nebulised saline and nebulised terbutaline as an adjunct to chest physiotherapy. Thorax 1988; 43(1):57–60. 3353875 10.1136/thx.43.1.57 PMC461097

[54] Poole PJ, Brodie SM, Stewart JM, Black PN. The effects of nebulised isotonic saline and terbutaline on breathlessness in severe chronic obstructive pulmonary disease (COPD). Australian and New Zealand journal of medicine 1998; 28(3):322–6. 9673744 10.1111/j.1445-5994.1998.tb01956.x

[55] Khan SY, O'Driscoll BR. Is nebulized saline a placebo in COPD? BMC pulmonary medicine 2004; 4:9 15458566 10.1186/1471-2466-4-9 PMC526282

[56] Cegla UH. Langzeittherapie über 2 Jahre mit Ambroxol (Mucosolvan) Retardkapseln bei Patienten mit chronischer Bronchitis. Ergebnisse einer Doppelblindstudie an 180 Patienten. Praxis und Klinik der Pneumologie 1988; 42(9):715–21. 3141915

[57] Olivieri D, Zavattini G, Tomasini G, Daniotti S, Bonsignore G, Ferrara G et al Ambroxol for the prevention of chronic bronchitis exacerbations: long-term multicenter trial. Protective effect of ambroxol against winter semester exacerbations: a double-blind study versus placebo. Respiration; international review of thoracic diseases 1987; 51 Suppl 1:42–51. 3299567 10.1159/000195274

