# Supplementary material for: Mucolytic Effectiveness of Tyloxapol in Chronic Obstructive Pulmonary Disease - A Double-Blind, Randomized Controlled Trial
Source: PLoS One. 2016 Jun 16;11(6):e0156999. doi: 10.1371/journal.pone.0156999 (PMC4911120; doi:10.1371/journal.pone.0156999)
Supplement: S1 Study Protocol — (PDF) [file pone.0156999.s003.pdf]

CLINICAL STUDY PROTOCOL

A double-blind, placebo controlled, randomized crossover trial to characterize the mucolytic effectiveness of tacholiquin ®.

A randomized double blind placebo controlled trial

Medaiumun 2014/1

Date of Final Protocol: 02.06.2014

Study Director: Prof. Dr. med. Stefan Zielen
Klinik für Kinder- und Jugendmedizin
Allergologie, Pneumologie und Mukoviszidose
Goethe-Universität
Theodor-Stern-Kai 7
60590 Frankfurt/Main
Tel.: 069/6301-83063,
Fax: 069/6301-83349
E-Mail: Stefan.Zielen@kgu.de

Sponsor: bene-Arzneimittel GmbH
Herterichstr. 1-3
D - 81479 München
T +49 89 74 987 - 232
F +49 89 74 987 - 233

Clinical Project Manager of Sponsor:	Dr. med. Gerald Untersteiner<br>bene-Arzneimittel GmbH<br>Herterichstr. 1-3<br>D - 81479 München<br>**T** +49 89 74 987 - 232<br>**F** +49 89 74 987 – 233<br>E-Mail: g.untersteiner@bene-gmbh.de
Biostatistician:	Frau Emilia Salzmann<br>Klinikum der Johann Wolfgang Goethe-Universität<br>Theodor-Stern-Kai 7<br>60590 Frankfurt/Main<br>E-Mail: Emilia.Salzmann@kgu.de
Study Site:	Medaimun GmbH<br>Kennedyallee 97 a<br>60596 Frankfurt<br>Tel: +49 (0) 69-69 59 58 90-0<br>Fax: +49 (0) 69-69 59 58 90-9<br>E-Mail: info@medaimun.de

1 OUTLINE TRIALS APPLICATION

1. STUDY SYNOPSIS

**COORDINATING INVESTIGATOR**	Prof. Dr. med. Stefan Zielen, Medaimun GmbH & Allergologie, Pneumologie und Mukoviszidose,Goethe-Universität; Theodor-Stern-Kai 7; 60590 Frankfurt Tel.: (069) 6301-83063; Fax (069) 6301 83349 E-mail: Stefan.Zielen@kgu.de
**TITLE OF STUDY**	A double-blind, placebo controlled, randomized crossover trial to characterize the mucolytic effectiveness of Tylaxopol, Tacholiquin ® in chronic bronchitis
**CONDITION**	Mucolytic treatment in chronic bronchitis
**OBJECTIVE(S)**	To evaluate the mucolytic activity of Tylaxopol compared to saline (0.9%) in chronic bronchitis patients. Lung function parameters, biomarker profiles in sputum and serum, and clinical symptoms by standardized questionnaires [COPD activity index (CAT), Baseline Dyspnea Index (BDI) & Transition Dyspnea Index (TDI)] will evaluated in response to Tylaxopolv. saline in chronic bronchitis patients.
**INTERVENTION (S)**	Chronic bronchitis patients (will be randomly assigned to either inhaled Tacholiquin®1% 5ml or saline solution each three times daily for 3 weeks. After one week of washout Tylaxopol treated patients will receive saline solution and vice versa for another 3 weeks.
**KEY INCLUSION AND EXCLUSION CRITERIA**	Key inclusion criteria: Patients aged between 40 and 85 years suffering from chronic bronchitis (COPD). Key exclusion criteria: Patients with other underlying chronic diseases.
**OUTCOME(S)**	The primary outcome variable is sputum weight after treatment as difference between that after Tylaxopol and after saline. Key secondary endpoint(s) are: symptom scores (CAT, BDI and TDI), ease of sputum production by analog scale, changes in lung function parameter (FVC, FEV1, MEF25, RV, RV/TLC), LCI, sputum parameters and biomarker profiles (IL-1, IL-6, IL-8), in sputum and response to Tylaxopolv saline. Assessment of safety: Safety will be ensured by monitoring of symptom scores and rescue medication, lung function FEV1 and adverse events.
**DURATION OF TREATMENT AND FOLLOW-UP**	Duration of treatment per treatment arms (3 weeks): Each patient will be treated in a cross-over double-blinded randomized design either with Tylaxopol and or saline. During each treatment arm is a one week washout phase.
**STUDY TYPE**	This study is a monocenter double-blind, placebo-controlled (DBPC) crossover randomised trial.

**STATISTICAL ANALYSIS**	The primary endpoint is sputum weight after 3 weeks of Tylaxopol– that after 3 weeks of saline treatment which will be analyzed for the Per Protocol Population. Estimated differences will be presented together with 95% confidence intervals (CIs) for the difference and two-sided p-values from a corresponding t-test. This analysis will be repeated for the ITT Population. Sample size is based on an expected difference in sputum weight of about 1.2 g corresponding to about 40% of that expected for saline with a standard deviation of s=1.7 g roughly corresponding to a correlation between sputum weights of ρ=0.75. Then a sample size of 20 achieves a power of 80%.<br>Secondary endpoints: The analysis of symptom scores ((CAT, BDI and TDI), ease of sputum production by analog scale, lung function parameter (FVC, FEV1, MEF25, RV, RV/TLC), will use the same methodology as that for the primary endpoint, using the ITT population. The analysis will include Day 1 and week 3 data. In addition acute changes of sputum weight on Day 1 at each treatment arm will be analyzed as described for the primary parameter.<br>Other Endpoints: Biomarker profiles (sputum cells, IL-1, IL-6, IL-8), in sputum) will be analyzed using an analysis of covariance (ANCOVA) model with covariates of baseline, smoking status and treatment. Estimated treatment differences along with corresponding 95 % CIs will be presented for the treatment comparison.
**SAMPLE SIZE**	To be assessed for eligibility: n = 26. To be allocated treatment: n = 20 for each treatment phase. To be analysed on per protocol basis: n=18
**TRIAL DURATION**	First patient in (FPI) to last patient out (LPO): FPI 1st August 2014. LPI: 30 Juni 2015. Duration of the entire trial: 12 months
**PARTICIPATING CENTER**	Medaimun GmbH ; Kennedyallee 97 a, 60596 Frankfurt, Germany

Study Flow Chart and Procedures

Period	I	II	III	IV	V	VI
	Treatment Phase A	Treatment Phase A	Treatment Phase A	Treatment Phase B	Treatment Phase B	Treatment Phase B
**Visit(s)**	1	2	3	4	5	6
	**Recruitment - Start of Treatment A**	**Treatment Visit**	**End of Phase I**	**Start of Treatment Phase B**	**Treatment Visit**	**End of Phase B**
**Weeks (W)**	**0**	**1**	**3**	**4**	**6**	**7**
**Day**	**0**	**7 ±2**	**21±4**	**28±14**	**7±2 after Visit 4**	**21±4 after Visit 4**
**Written Informed consent**	X					
**Demographic data**	X					
**Medical/COPD history and concomitant diseases**	X					
**Smoking History/Status**	X					
**Concomitant medications**	X	X	X	X	X	X
**Inclusion/ Exclusion**	X					
**Physical Examination Vital Signs**	X	X	X	X	X	X
**Lung function (Bodyplethys.)**	X	X	X	X	X	X
**Post Reversibility Lung function**	X	X	X	X	X	X
**Urine Pregnancy testing[1]**	X	X	X	X	X	X
**Laboratory examinations [2]**	X					
**COPD Assessment Test (CAT) handover**	X	X	X	X	X	X
**Collection and Review of BDI / TDI St George**	X		X		X	X

**Inhalation of medication**	X	X	X	X	X	X
**Lung function after Sputum collection**	X	X	X	X	X	X
**Monitoring Ease of expectoration**	X	x	x	x	x	x
**Nebulizer and Drug dispensation**	X			X		
**Collection of trial drug/ Compliance check**	x	x	X	x	x	X
**Recording of Adverse Events**		X	X	X	X	X
**Appointment for the next visit**	X	X	X	X	X	

1) If applicabel
2) Laboratory examinations : Hematology Makrophagen Activation test

TABLE OF CONTENTS

STUDY PERSONNEL .... FEHLER! TEXTMARKE NICHT DEFINIERT.

FLOW CHART 6

LIST OF ABBREVIATIONS 11

1 BACKGROUND AND RATIONALE FOR THE STUDY 12

1.1. Tacholiquin® .... **FEHLER! TEXTMARKE NICHT DEFINIERT.**
1.2. EXPERIENCE WITH TACHOLIQUIN® 13
1.3. TOLARANCE OF TACHOLIQUIN® .... **FEHLER! TEXTMARKE NICHT DEFINIERT.**
1.4. RATIONALE 15

2 OBJECTIVE OF THE STUDY AND ENDPOINTS 15

2.1. OBJECTIVE OF THE STUDY 15
2.2. PRIMARY ENDPOINT 15
2.3. SECONDARY ENDPOINTS 15
2.4. EXPLORATIVE ENDPOINTS 156
2.5. SAFETY .... **FEHLER! TEXTMARKE NICHT DEFINIERT.**6

3 STUDY DESIGN 166

4 STUDY POPULATION 166

4.1. INCLUSION CRITERIA 167
4.2. EXCLUSION CRITERIA 16
4.3. WITHDRAWAL CRITERIA 188

5 STUDY MEDICATION 18

5.1. INVESTIGATIONAL DRUG 18
5.2. COMPARATOR DRUG (PLACEBO) 19
5.3. DOSAGE AND TREATMENT SCHEDULE 19
5.4. SUPPLY, LABELLING AND PACKAGING ARRANGEMENTS 19
5.5. METHODS OF BIAS REDUCTION 190
5.5.1. Randomisation 190
5.5.2. Blinding 200
5.5.3. Emergency code 20
5.6. TREATMENT ASSIGNMENT 201
5.7. STORAGE CONDITIONS 20
5.8. CONCOMITANT MEDICATIONS 21
5.8.1. AUTHORIZED MEDICATIONS INCLUDING RESCUE MEDICATION 21
5.8.2. Unauthorized medications .... ***Fehler! Textmarke nicht definiert.***

6 STUDY PROCEDURES AND ASSESSMENTS 212

6.1. STUDY PROCEDURE 212
6.1.1. Recruitment procedure 21
6.1.2. Demographic data, baseline data 22
6.2. PROCEDURE BY VISIT 22
6.2.1. METHODS AND MEASUREMENTS 22
6.2.2. Instructions 255
MEDICAL HISTORY 25
PHYSICAL EXAMINATION AND VITAL SIGNS 25

LABORATORY EXAMINATIONS ... 266
PULMONARY FUNCTION-TESTING ... 27
CLINICAL SYMPTOM SCORES ... 27
6.3. REPLACEMENT OF DROP-OUTS AND WITHDRAWALS ... 28

7 ASSESSMENT OF EFFICACY AND SAFETY ... 28

7.1. ASSESSMENT OF EFFICACY ... 28
7.1.1. Primary efficacy variable ... 28
7.1.2. Secondary efficacy variables ... 29

7.2. ASSESSMENT OF SAFETY ... 290
7.3. LABORATORY ASSESSMENTS ... 290

8 ADVERSE EVENTS ... 300

8.1. DEFINITION 1 ... 30
8.2. DEFINITION 2 ... 311
8.3. DEFINITION 3 ... 321
8.4. REPORTING AND RECORDING OF ADVERSE EVENTS ... 33
8.5. REPORTING, RECORDING AND HANDLING OF SERIOUS ADVERSE EVENTS (SAES) ... 33

9 DATA MANAGEMENT AND STATISTICS ... 34

9.1. DATA MANAGEMENT ... 34
9.2. BLIND REVIEW ... 34
9.3. SAMPLE SIZE CALCULATION ... 34
9.4. ANALYSIS OF EFFICACY CRITERIA ... 35
9.5. EXPLORATORY ANALYSIS OF EFFICACY CRITERIA ... 35
9.6. ANALYSIS OF SAFETY DATA ... 35
9.7. ANALYSED POPULATION ... 35

10 ETHICAL CONSIDERATIONS AND PATIENT'S CONSENT ... 36

10.1. ETHICAL CONSIDERATIONS ... 36
01.3. INFORMED CONSENT ... 36

11 INSURANCE ... 37

12 PATIENT DOCUMENTATION AND MONITORING ... 377

13.1. CASE REPORT FORM (CRF) ... 37

13 DRUG ACCOUNTABILITY ... 37

14 ADMINISTRATIVE ISSUES ... 38

15.1. RULES FOR AMENDING THE PROTOCOL ... 38
15.2. DISCONTINUATION OF THE TRIAL BY THE SPONSOR ... **FEHLER! TEXTMARKE NICHT DEFINIERT.**
15.3. ARCHIVING ... 38

15 STATEMENT OF CONFIDENTIALITY ... 37
SECRECY AGREEMENT ... 39

17 REPORTING AND PUBLICATION ... 38

18 FINANCIALS ... 38

19 CONTINUATION SUPPLY ... 39

20 SCIENTIFIC AGREEMENT / SIGNATURES ... 39

SIGNATURES PAGE **40**

22 REFERENCES **41**

LIST OF ABBREVIATIONS

AE	Adverse Event
ATS	American Thoracic Society
BC	Blood count
CAT<br>CBA	COPD Activity Index<br>Cytometric Bead Array
COPD	Chronic Bronchitis
CRFs	Case Report Forms
IL-6	Interleukin 6
FEV_1	Forced Expiratory Volume in 1 second
FVC	Forced Vital Capacity
GCP	Good Clinical Practice
L	Liter
Mg	Milligrams
RBC	Red Blood Cell
RV	Residual volume
SABA	Short-acting beta-2-agonists
SAE	Serious Adverse Event
SD	Standard deviation
SUSAR	Suspected, Unexpected, Serious Adverse drug Reaction
RV/TLC	Functional residual capacity
WBC	White Blood Cell

1 BACKGROUND AND RATIONALE FOR THE STUDY

In the classical phenotype of chronic bronchitis, mucus hypersecretion is the key presenting symptom and its contribution to airflow obstruction and has been the subject of debate for a long time (1). By the 1950s it was known that tobacco smoking was associated with chronic cough and sputum production, and that smokers who developed chronic bronchitis had impaired lung defences, a condition favouring colonisation and infection of the lower airways (2). Biopsies showed that the excess mucus production which defined chronic bronchitis was associated with enlarged bronchial glands and that an approximate relationship existed between the presence of chronic bronchitis and emphysema (3). It is well established that chronic mucus hypersecretion is significantly and consistently associated with both an increase in forced expiratory volume in 1 s (FEV1) decline and an increase of subsequent hospitalisation (4-6).

A large number of studies have been performed on the use of mucolytic drugs in the treatment of chronic bronchitis and chronic obstructive bronchitis (COPD). The outcomes have been reviewed in several meta-analyses (7, 8). Overall, there was a significant reduction in exacerbations (0.05 per patient per month) and the number of days with disability (0.56 days per patient per month). Mucolytics were well tolerated and the number of adverse events was lower than with placebo (7).

The aim of the present study is to assess the effects of the “old” mucolytic agent, Tacholiquin ® compared to saline (0.9%) in patients with chronic bronchitis (COPD). Tacholiquin ® -Tyloxapol is a polymeric covalent compound with multiple mucolytic actions. Tyloxapol is a product which has been developed in Europe in the fifties; therefore the standards used for its development are partially different to those currently applied. Despite extensive clinical evidence of its clinical usefulness little is known about the mucolytic activity of Tyloxapol compared to saline solution.

- There are no randomized double-blind, placebo controlled trials available demonstrating possible superiority of Tyloxapol vs. saline.
- No data exists of mucolytic action on quality of life and lung function parameters in chronic bronchitis
- No specific studies are available in human investigating biomarker profiles (in sputum and serum after Tyloxapol inhalation

1.1. EXPERIENCE WITH TYLAXOPOL

Tacholiquin ® -Tyloxapol is a polymeric covalent compound of the ethylene oxide ether of an octyl-phenol-formaldehyde condensation product). The active substance acts in a neutral manner in relation to acids, bases and salts. Tyloxapol can be heated to a temperature necessary for sterilisation without impairing its physical properties.

Tyloxapol has been used therapeutically for over 40 years and has proved to be well tolerated during this time (9-16). Tyloxapol influences the respiratory system with the following three different action mechanisms:

- Secretolytic action
- Reduction of surface tension
- Dissolution of coatings
- Down regulating inflammation.

1.1.1 Secretolytic action

In an experiment, Tainter et al. (17) proved that small quantities of tyloxapol applied in aerosol form liquefy the sputum. According to Ravenel (15), the viscosity of the sputum is reduced by 10% to 20% by adding tyloxapol. Wilde (18) examined the effect of tyloxapol on surface tension and viscosity of sputa in vitro. When measuring viscosity with rotational viscosimetry, Wilde established that: "The longer tyloxapol acts on the mucous content and the higher the volume content, which is likely to go hand in hand with inhalation of tyloxapol, the thinner the sputum becomes" (18). In a double-blind crossover study of 20 patients, Paez et al. (19) compared the ability of sodium chloride solution, distilled water, sodium ethasulfate and tyloxapol with regard to their ability to act secretolytically. The test subjects were 20 patients with chronic obstructive pulmonary diseases. In aerosol form, tyloxapol led to a significant increase in sputum volume and sputum dry weight (by 48.6% or 84 mg/h) compared with distilled water (19).

1.1.2 Reduction of surface tension

Hutschenreuter showed that tyloxapol reduces surface tension (9). Wilde (18) also examined the influence of tyloxapol on the surface tension of sputum. He established that after adding tyloxapol before rotational viscosimetry, the surface tension values of the sputa fell significantly, namely up to ¾ of their initial value.

1.1.2 Reduction of surface tension

Farber et al. (20) showed that tyloxapol also penetrates the mucous wall. Through moistening of the accumulations of mucus and mucus plugs, tyloxapol dissolves the viscous and dried secretion from the layer underneath it, thus enabling normal ciliary activity in the respiratory tracts.

1.1.3 Down regulation of inflammation

Tyloxapol inhibits activation of the transcription factor nuclear factor-kappa B (NK-kappa B), reduces resting secretion of the cytokine interleukin-8 (IL-8) in cultured human monocytes, and inhibits pro-inflammatory cytokines like lipopolysaccharide (LPS)-stimulated release of tumor necrosis factor-alpha (TNF-alpha), IL-1 beta, IL-6, IL-8, granulocyte-macrophage colony-stimulating factor (GM-CSF), and the eiconsanoids thromboxane A2 and leukotriene B4 (21).

1.2. TOLARANCE OF TYLAXOPOL

Side-effects in the form of hypersensitivity reactions have only occurred very rarely. Nausea has occasionally occurred in allergic subjects. Occasionally, the first deep breaths may be followed by an initial urge to cough, which immediately disappears after moistening of the mucous membrane.
Hutschenreuter administered more than 1000 individual inhalations of Tacholiquin ® ® to 106 surgical patients. In doing so, he showed that Tacholiquin ® was very well tolerated by the patients. There were also no signs of intolerance in patients who inhaled tyloxapol several times a day over a period of 9 weeks (9).
In various studies, patients were treated continuously with tyloxapol for up to 4 years (in one case, it was administered in an aerosol tent every night for 4 years) with no side-effects being observed (10, 17, 22).
Segal et al (23) administered Tacholiquin ® continuously over 24 hours per day to children and adults with pertussis over a period of 7 days, and observed no side-effects (23).
Farber et al. (20) pointed out that tyloxapol is harmless, safe and effective in the treatment of patients with bronchiectases. According to the authors, tyloxapol facilitates the expectoration of sputum. In particular, the authors pointed out that tyloxapol caused no serious side-effects and is an ideal carrier for antibiotics, bronchodilators and substances with a vasoconstrictive action as a result of its chemical structure.
Camarata et al. (24) also established no adverse drug reactions after this active substance was administered to patients with atelectases.
There are no known interactions with other drugs.

1.3. RATIONALE

The aim of the study is to assess the mucolytic effects of tyloxapol compared to saline (0.9%) in patients with chronic bronchitis (COPD).

2 OBJECTIVE OF THE STUDY AND ENDPOINTS

2.1. OBJECTIVE OF THE STUDY

Determine the magnitude of the effect of tyloxapol by means of lung function parameters, biomarker profiles in sputum and serum, and clinical symptoms and quality of life by standardized questionnaires [COPD activity index (CAT), Baseline Dyspnea Index (BDI) & Transition Dyspnea Index (TDI), St George's respiratory Quality of Life Questionnaire] will evaluated in response to tyloxapol ® vs. saline at day one and at end of treatment.

2.2. PRIMARY ENDPOINT

The primary endpoint is sputum weight after 3 weeks of Tylaxopol– that after 3 weeks of saline treatment which will be analyzed for the Per Protocol Population. Estimated differences will be presented together with 95% confidence intervals (CIs) for the difference and two-sided p-values from a corresponding t-test.

2.3. SECONDARY AND EXPLORATIVE ENDPOINTS

Secondary endpoints:

The analysis of symptom scores ((CAT, BDI,TDI and St George's respiratory Quality of Life Questionnaire), ease of sputum production by analog scale, lung function parameter (FVC, FEV1, MEF25, RV, RV/TLC), LCI will use the same methodology as that for the primary endpoint, using the ITT population. The analysis will include Day 1 and week 3 data. In addition acute changes of sputum weight on Day 1 at each treatment arm will be analyzed as described for the primary parameter.

Explorative endpoints:

Biomarker profiles (sputum cells, IL-1, IL-6, IL-8), in sputum will be analysed using an analysis of covariance (ANCOVA) model with covariates of baseline, smoking status and treatment. Estimated treatment differences along with corresponding 95 % CIs will be presented for the treatment comparison.

In addition anti-inflammatory activity of tyloxapol will be analyzed in vitro by the macrophage activation test.

3 STUDY DESIGN

The study will be a monocentre, randomized, double-blind, crossover, placebo-controlled study in 24 COPD patients (including 4 Drop-out) in order to achieve 20 evaluable patients for the statistical analysis. 'Evaluable' mean eligible COPD patients having met all the inclusion and none of the exclusion criteria, having performed all scheduled clinical visits and with a “good” compliance as defined in section 7.5.

4 STUDY POPULATION

4.1. INCLUSION CRITERIA

For inclusion in the COPD patients should fulfill the following criteria:

1. Provision of informed consent prior to any study specific procedures.
2. Female or male subjects aged 40-85 years inclusive at Visit 1.
3. Documented history COPD with a post-bronchodilator FEV1/FVC<0.70 and a post-bronchodilator FEV1<80% of predicted normal value at screening (spirometry will be used for this criteria assessment).
4. Current smoker or ex-smoker with a tobacco history of ≥10 pack-years (1 pack year = 20 cigarettes smoked per day for 1 year).
5. Women of childbearing potential (WOCBP) must use a highly effective form of birth control (confirmed by the Investigator).

− Women <50 years old would be considered postmenopausal

6. At least a CAT value > 10 at Visit 1.

4.2. EXCLUSION CRITERIA

1. Clinically important pulmonary disease other than COPD (e.g. active lung infection, clinically significant bronchiectasis, pulmonary fibrosis, cystic fibrosis, hypoventilation syndrome associated with obesity, lung cancer, alpha 1 anti-trypsin. deficiency and

primary ciliary dyskinesia) or another diagnosed pulmonary or systemic disease that is associated with elevated peripheral eosinophil counts (e.g. allergic bronchopulmonary aspergillosis/mycosis, Churg-Strauss syndrome, hypereosinophilic syndrome).

2. Female Any disorder, including, but not limited to, cardiovascular, gastrointestinal, hepatic, renal, neurological, musculoskeletal, infectious, endocrine, metabolic, haematological, psychiatric, or major physical impairment that is not stable in the opinion of the Investigator and could:

− Affect the safety of the subject throughout the study

− Influence the findings of the study or their interpretation

− Impede the subject's ability to complete the entire duration of study

3. Documented Unstable ischemic heart disease, arrhythmia, cardiomyopathy, heart failure, renal failure, uncontrolled hypertension as defined by the Investigator, or any other relevant cardiovascular disorder as judged by the Investigator that in Investigator's judgment may put the patient at risk or negatively affect the outcome of the study.

4. Treatment with systemic corticosteroids and/or antibiotics, and/or hospitalizationfor a COPD exacerbation within 4 weeks prior to (Visit 1)

5. Acute upper or lower respiratory infection requiring antibiotics or antiviral medication within 4weeks prior to (Visit 1)

6. Pneumonia within 4 weeks prior to (Visit 1), based on the last day of antibiotic treatment or hospitalization date, whatever occurred later. The subject cannot be re-screened if this exclusion criterion is met.

7. History of anaphylaxis to Tacholiquin ®.

8. Long term oxygen therapy (LTOT) with signs and/or symptoms of cor pulmonale, right ventricular failure or evidence by echocardiogram or pulmonary artery catheterization of moderate to severe pulmonary hypertension. In order to be admitted to the trial subjects on LTOT have to be ambulatory and be able to attend clinic visits.

9. Any clinically significant abnormal findings in physical examination, vital signs, haematology, or urinalysis during Visit 1 , which, in the opinion of the Investigator, may put the subject at risk because of his/her participation in the study, or may influence the results of the study, or the subject's ability to complete entire duration of the study.

10. Use of immunosuppressive medication, including rectal corticosteroids, high potency topical corticosteroids and systemic steroids within 28 days prior to (Visit 1).

4.3. WITHDRAWAL CRITERIA

Reasons that a patient may discontinue participation in a clinical study are considered to constitute one of the following:

1. Occurrence of serious adverse drug reactions (at least in suspected relationship with study medication) or of other intolerable adverse events, as judged by the investigator
2. Development of any pathology, condition or surgery defined in the exclusion criteria (major protocol violation).
3. Protocol violation
4. Pregnancy
5. Subject who withdraw consent
6. Lost to follow-up
7. Administrative problems
8. Death

The reason for withdrawal must be reported on the CRF “Terminal Study Status” page and all study medication have to be returned. For withdrawn patient a full examination according to the last planned visit should be performed, whenever possible.

5 STUDY MEDICATION

5.1. INVESTIGATIONAL DRUG

Tacholiquin ® contains:
1% (0.1%) tyloxapol
5% glycerine
2% sodium hydrogen carbonate in a sterile aqueous solution.

Tacholiquin ® will be provided as 1% solution and inhaled via nebulizer three times daily. The preparation is available as 5 ml syringe stable for 4 weeks. Matching syringes with 5 ml 0.9% Saline will be used as placebo.

5.2. COMPARATOR DRUG

Matched syringes with 5 ml 0.9% Saline will be used as comparator drug.

5.3. DOSAGE AND TREATMENT SCHEDULE

The dosage regimen will be three inhalations with 5 ml solution via nebulizer per day of study treatment during 21 consecutive days. Patients will be randomized either starting with Tacholiquin ® or Saline 0.9% in treatment phase A. After treatment phase A patients will receive the alternative drug in treatment phase B.

5.4. SUPPLY, LABELLING AND PACKAGING ARRANGEMENTS

All study medication will be supplied to the principal investigator by benepharma via the Central Apotheke Steinbach. The central Apotheke will deliver to the study site syringes with study treatment.

For each patient syringes with Tylaxopolor placebo will be provided for the double-blind treatment period (including 6 reserve syringes). The study medication is presented in box wallets, each containing 3X 21 syringes (for 21 days of treatment).

The patients will have to bring back their study medication package (including reserve medication) for compliance check at Visit 3 and 6.

5.5. METHODS OF BIAS REDUCTION

5.5.1. RANDOMISATION

Randomisation will be performed by Prof. Herrmann using a validated system (RandList 2.0) that automates the random assignment of treatment groups to randomisation numbers.

Treatments are randomly allocated to patients, according to the random permuted block scheme. The randomisation number will have 2 digits, starting at 01.

In accordance with the ICH Biostatistics Guideline, the block size is intentionally not given in the study protocol. The generation of the random code list including the production of sealed envelopes is performed in a validated environment.

5.5.2. BLINDING

The study is conducted as a double-blind study.

For the treatment period, the syringes with 5 ml will not differ in appearance (concerning form, weight, color, texture of content, etc.), to ensure patient and investigator blinding.

The investigators and personnel involved in study will remain blinded throughout all periods of the study, except in the case of an emergency. Study drug codes will not be available to the above personnel until completion of the clinical trial report.

5.5.3. EMERGENCY CODE

The Investigator will be provided with a sealed envelope containing the code for each patient's randomisation number. The code can only be broken under serious adverse event circumstances. If a code-break occurs, the investigator will record the reason for the code-break, the date of opening and will sign the envelope in the appropriate place, withdrawing the patient from the study. The investigator will communicate the event of breaking the code to the Sponsor.

5.6. TREATMENT ASSIGNMENT

At Visit 1 (Day 0) eligible patients will be assigned a randomization number in consecutive, ascending order.

In order to ensure random allocation, each new consecutive patient randomized is to be given the box bearing the lowest available randomisation number.

The investigator must maintain an accurate record of the shipment and dispensing of study drug in a drug accountability log. An accurate record of the date and amount of study drug dispensed to each patient and returned must be available for inspection at any time.

The investigator will be asked to return all unused medication at the end of the study.

Volunteers are required to be dosed so that the time window between visits is within 7 days of the scheduled administration dates.

5.7. STORAGE CONDITIONS

Syringes either with Tylaxopolor saline 0.9% have to be stored at room temperature (not above 25°).

Drug supplies must be kept in an appropriate, secure area (e.g. locked cabinet) and stored according to the conditions specified on the drug labels.

5.8. CONCOMITANT MEDICATIONS

All concomitant medication must be recorded in the CRF on the concomitant medication pages at each visit. Patient diary will also be check at each visit verify if patient has an AE or has taken concomitant medications.

5.8.1. AUTHORIZED MEDICATIONS INCLUDING RESCUE MEDICATION

Use of ß-2 agonists (salbutamol 100 µg 2 puffs) is allowed for the treatment of symptoms during the study. Each nebulization of salbutamol should be recorded on the patient's diary and will be included in the analysis of medication use.

6 STUDY PROCEDURES AND ASSESSMENTS

6.1. STUDY PROCEDURE

The study design is shown in Fig: 1.

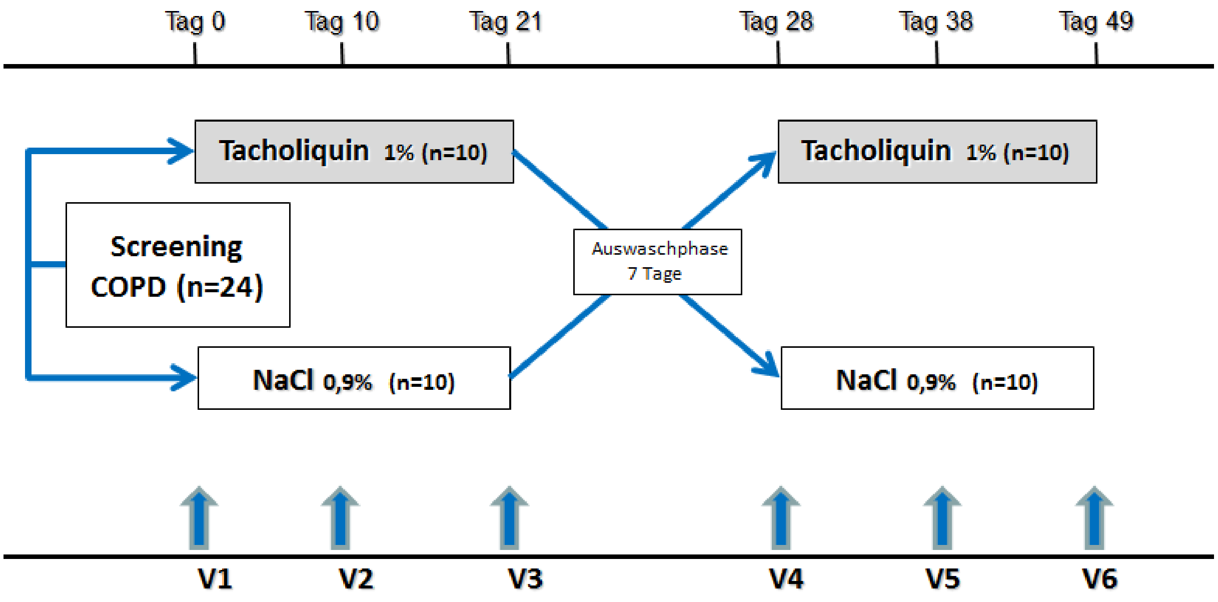

6.1.1. RECRUITMENT PROCEDURE

At Visit 1 before any assessment necessary for the study, the COPD patient will be informed by the physician according to the “Patient information” sheet. The patient will then give a written consent by signing the Patient Consent. As soon as the specific inclusion and exclusion criteria at Visit 1 have been checked, eligible patients will be assigned a randomization number in ascending order. They will then start double-blind trial treatment.

Additional visits can be scheduled and performed according to the investigators judgment of how to best manage the patient. At these visits in addition to recording any SAEs, AEs and concomitant medication use, a physical examination, vital sign assessment and lung function tests are to be performed.

Any unscheduled visits are to be recorded in the other clinical visits CRFs.

6.1.2. DEMOGRAPHIC DATA, BASELINE DATA

Age, sex, ethnic origin, height, weight, smoking status, general medical history, past and concomitant medication, clinical status, allergic history, lung function at the initial visit are to be reported to check the homogeneity of the sample.

6.2. PROCEDURE BY VISIT

6.2.1. METHODS AND MEASUREMENTS

Visit procedures at screening visit and before treatment phase A (Visit 1)

- Patient information/Informed consent
- Date of visit
- Demographic data,
- Medical history and concomitant diseases
- Physical examination / Vital signs
- Concomitant medications
- Bodyplethysmograhy
- Bronchodilator response to Salbutamol
- Completing Questionnaires (CAT, BDI, St George)
- Inclusion/exclusion criteria

- Randomisation and drug dispensation
- Laboratory examination (including Urinalysis)
- Inhalation of blinded medication by nebuliser
- Sputum collection
- Recording side effects
- Recording “ease of expectoration”.
- Lung function monitoring up to one hour after sputum collection
- Lung clearance index one hour after inhalation
- Dairy explanation
- Appointment for the next visit

Visit procedures at visit (Visit 2, day 7 ± 2 days)

- Date of visit
- Physical examination / Vital signs
- Concomitant medications
- Bodyplethysmograhy
- Bronchodilator response to Salbutamol
- Completing Questionnaires (CAT)
- Inhalation of blinded medication by nebuliser
- Sputum collection
- Recording side effects
- Recording “ease of expectoration”.
- Lung function monitoring up to one hour after sputum collection
- Drug dispensation
- Recording of Adverse Events
- Appointment for the next visit

Visit procedures after end of treatment phase A at visit (Visit 3, day 21 ± 4 days)

- Date of visit
- Physical examination / Vital signs
- Concomitant medications
- Bodyplethysmograhy
- Bronchodilator response to Salbutamol
- Completing Questionnaires (CAT and TDI, and St. George)
- Inhalation of blinded medication by nebuliser
- Sputum collection
- Recording side effects

- Recording “ease of expectoration”.
- Lung function monitoring up to one hour after sputum collection
- Appointment for the next visit after “Wash-out –phase”.

Visit procedures after washout (+ 7-14 days) before treatment phase B at visit 4

- Date of visit
- Physical examination / Vital signs
- Concomitant medications
- Bodyplethysmograhy
- Bronchodilator response to Salbutamol
- Completing Questionnaires (CAT, BDI and St. George)
- Drug dispensation
- Inhalation of blinded medication by nebuliser
- Sputum collection
- Recording side effects
- Recording “ease of expectoration”.
- Lung function monitoring up to one hour after sputum collection
  Appointment for the next visit

Visit procedures at visit 5 (day 7 ± 2 days after Visit 4)

- Date of visit
- Physical examination / Vital signs
- Concomitant medications
- Bodyplethysmograhy
- Bronchodilator response to Salbutamol
- Completing Questionnaires (CAT)
- Inhalation of blinded medication by nebuliser
- Sputum collection
- Recording side effects
- Recording “ease of expectoration”.
- Lung function monitoring up to one hour
- Drug dispensation
- Recording of Adverse Events
- Appointment for the next visit

Visit procedures at visit 6 end of treatment phase B (21 ± 4 days after Visit 4)

- Date of visit
- Physical examination / Vital signs
- Concomitant medications
- Bodyplethysmograhy
- Bronchodilator response to Salbutamol
- Lung clearance index
- Completing Questionnaires (CAT, TDI and St. George)
- Drug dispensation
- Inhalation of blinded medication by nebuliser
- Sputum collection
- Recording side effects
- Recording “ease of expectoration”.
- Lung function monitoring up to one hour after sputum collection

Additional visits can be scheduled and performed according to the investigators judgment of how to best manage the patient. At these visits in addition to recording any SAEs, AEs and concomitant medication use, a physical examination, vital sign assessment and lung function tests will be performed.

Any unscheduled visits, follow-up visits and adverse events, are to be recorded in the other clinical visits CRFs.

6.2.2. INSTRUCTIONS

MEDICAL HISTORY

At Visit 1, medical history, and concomitant medication according to the CRF is obtained.

PHYSICAL EXAMINATION AND VITAL SIGNS

A physical examination is performed by the investigator at each visit.

The physical examination should include the following:

- General appearance
- Skin
- O2 Saturation
- Head
- Respiratory (lungs)
- Cardiovascular
- RR, pulse
- Musculoskeletal

LABORATORY EXAMINATIONS

A venous blood is obtained at the following visits (Visit 1). An urine sample is analyzed by “Chek-stix Combo” at visit 1.

Macrophage activation test (MAT)

The anti-inflammatory effect of Tyloxapol was measured by cytokine release after LPS-stimulation of whole blood cells. Briefly, EDTA-blood samples were diluted 1:10 with RPMI 1640 medium and stimulated with 5 ng/ml LPS from E. coli 026:B6 (Sigma-Aldrich Pharmaceuticals, Germany) in the presence of 0, 0.001, 0.01, 0.1 and 1.0 mg/ml Tyloxapol [22]. After a 24-hr incubation period at 37°C and 5% CO2, supernatants were harvested and stored at -80°C until further use.

Venous blood is sent to the laboratory of Prof. Schubert.

URINE PREGNANCY TESTS

Pregnancy tests are obtained from all female volunteers of child-bearing potential. At Visit 1, a urine pregnancy test is obtained.

PULMONARY FUNCTION-SPIROMETRY

Pulmonary function tests will be recorded whilst the subject is in a sitting position at every visits. Bodyplethysmography will be performed according to the European Respiratory Standards (ERS).

The following variables will be documented: FVC [L, % pred], FEV1 [L, % pred], MEF25 [L, % pred], FEV1/FVC (Tiffenau-Index), RV and RV/TLC.

In order to reduce variability, all measurements should be made at approximately the same time of day at each visit and on the same calibrated bodyplethysmography..

The FVC, FEV1, MEF 25%, FEV1/FVC, RV and RV/TLC recorded in the CRF must be taken from the maneuver obtained from the single “best test” curve and must be recorded as absolute values. The “best test” curve is defined as the spirogram that gives the largest FEV_1.

Between treatment differences for FEV_1, FVC and MEF 25% RV and RV/TLC will be analyzed at each visit using an analysis of covariance with pooled center, baseline, sex Least square mean differences based on the model will be calculated and 95% confidence intervals will be constructed.

PATIENT SYMPTOMS AND QUALITY OF LIFE

Assessment of Symptoms

Clinical symptoms will be assed by the COPD activity test (CAT) at each visit.

The possible improvement in dyspnoea will be assesed via the Baseline and Transition Dyspnoea Index (BDI/TDI) after each treatment phase. The BDI/TDI provides a multidimensional measurement of dyspnea based on 3 components that evoke dyspnea in activities of daily living, in symptomatic COPD patients.

Quality of life

Quality of life measures were made using the St George’s respiratory Quality of Life Questionnaire. Self-completed questionnaires were performed before and after each treatment phase. A clinically relevant change was considered to be 4 for total score. End-points included the global score change during active and placebo phases, as well as scores for the sub-scales of symptoms, activity and impact.

Ease of expectoration

Patients were asked to report their subjective view of the sputum ease of expectoration using a visual analogue scale after sputum expectoration as described. (25).This data was collected at all visits.

Patient diary

Patients are advised to keep a diary the study. Data will be included into a paper diary**.** Concomitant medication and adverse event will be recorded in the diary daily.

6.3. REPLACEMENT OF DROP-OUTS AND WITHDRAWALS

It will be documented whether or not each patient completed the clinical study. If for any patient either study treatment were discontinued the reason will be recorded. Patients terminating the study prematurely will not be replaced. An estimation of 4 patients dropping out of the study has been considered when determining the number of patients to be recruited.

7 ASSESSMENT OF EFFICACY AND SAFETY

7.1. ASSESSMENT OF EFFICACY

7.1.1. PRIMARY EFFICACY VARIABLE

The primary endpoint is sputum weight after 3 weeks of Tylaxopol and that after 3 weeks of saline treatment which will be analyzed for the Per Protocol Population. Estimated differences will be presented together with 95% confidence intervals (CIs) for the difference and two-sided p-values from a corresponding t-test. This analysis will be repeated for the ITT Population.

7.1.2. SECONDARY EFFICACY VARIABLES

Secondary endpoints: The analysis of symptom scores ((CAT, BDI and TDI), ease of sputum production by analog scale, lung function parameter (FVC, FEV1, MEF25, RV, RV/TLC), will use the same methodology as that for the primary endpoint, using the ITT population. The analysis will include Day 1 and week 3 data. In addition acute changes of sputum weight on Day 1 at each treatment arm will be analyzed as described for the primary parameter.

Other Endpoints: Biomarker profiles (sputum cells, IL-1, IL-6, IL-8), in sputum and serum (IL-6, LBP, CRP) will be analyzed using an analysis of covariance (ANCOVA) model with covariates of baseline, smoking status and treatment. Estimated treatment differences along with corresponding 95 % CIs will be presented for the treatment comparison

7.2. ASSESSMENT OF SAFETY

Safety assessments will consist of monitoring and recording all adverse events and serious adverse events, the regular monitoring of hematology, regular measurement of vital signs and the performance of physical examinations.

7.3. LABORATORY ASSESSMENTS

7.3.1. SPUTUM PROCESSING

Induced sputum will be analyzed for cell differentiation, as well as protein levels of cytokines.

Sputum will be sampled and processed as follows:

Subjects first inhale 200 µg Salbutamol and consecutively nebulised the study medication. During this procedure, it will be important to flush and clean the nose for disposal of lower part of squamous epithelium cells in the samples. Sputum will be processed within 2 hours of collection. The selected sputum plugs should be as far as possible without saliva, processed into a weighed Eppendorf tube and processed with 4x weight/volume of 0.1% Dithiothreitol. Afterwards 2x weight/volume of Phosphate-buffered saline (PBS) was added. Samples will be filtered through 48 µm mesh and centrifuged for 10 minutes at 790 x g to remove the cells. Supernatants will

be stored at -80C° until further analysis with Cytometric Bead Array (CBA) as described (26).

Sputum cells

Specimens containing levels of squamous epithelial cells less than 10% of the total inflammatory cell number will be considered adequate. At least 400 inflammatory cells will be microscopically counted for each specimen. Neutrophils, eosinophils, lymphocytes, basophils and macrophages will be expressed as percentages of the total cell count.

Cytometric bead array

For analyses of sputum and cell culture supernatants we will use the sandwich-ELISA based technique of Cytometric Bead Array (CBA) to detect different pro-inflammatory cytokines or chemokines according to the manufacturer's protocol. Concentrations of cytokines/chemokines will be determined in sputum samples or cell culture supernatants using the BD™ CBA Flex Set System for the measurement of IL-1, IL-6, IL-8, INF-ϒ, TNF-α and MCP-1, respectively. (BD Bioscience-PharMingen, San Diego, CA, USA) (26).

8 ADVERSE EVENTS

8.1. DEFINITION 1

Adverse Event (AE): an adverse event (AE) is any untoward medical occurrence in a patient or clinical trial subject administered a medicinal product and which does not necessarily have a causal relationship with this treatment. An adverse event can therefore be any unfavorable and unintended sign (including abnormal laboratory findings), symptom, or disease temporally associated with the use of an investigational medicinal product, whether or not considered related to the investigational medicinal product.

Adverse Drug Reaction (ADR): an adverse drug reaction is a response to a medicinal product, which is noxious and unintended and which occurs at doses normally used in man for the prophylaxis, diagnosis or therapy of disease or for the restoration, correction or modification of physiological function (article 1(11) of Directive 2001/83/EC).

All adverse events judged by either the reporting investigator or the sponsor as having a reasonable causal relationship to a medicinal product is qualified as adverse drug reactions. The expression reasonable causal relationship means to convey in general that there is evidence or argument to suggest a causal relationship.

Unexpected adverse event (UAE): an unexpected adverse event is an adverse event with the investigational product not previously reported (in nature, severity or incidence) and not consistent with the applicable product information (e.g. Investigator's Brochure for an unapproved investigational product or Summary of Product Characteristics (SPC) for an authorized product). If there are reasonable grounds for the suspicion that the UAE is causally related to the trial drug, it must be considered as ADR.
• When the outcome of the adverse event is not consistent with the applicable product information this adverse reaction should be considered as unexpected.
• Severity: the term “severe” is often used to describe the intensity (severity) of a specific event. This is not the same as “serious,” which is based on patient/event outcome or action criteria.

The adverse events intensity is classified using the following definitions:

- Mild: The adverse event is transient, requires no treatment, and does not interfere with the study’s subject daily activities.
- Moderate: The adverse event introduces a low level of inconvenience or concern to the study subject and may interfere with daily activities, but is usually ameliorated by simple therapeutic measures.
- Severe: The adverse event interrupts the study subject’s usual daily activity and requires systematic therapy or other treatment.

8.2. DEFINITION 2

Serious Adverse events: An adverse event (AE) is defined as serious whenever the outcome is:

a. death

b. requiring inpatient hospitalisation or prolongation of existing hospitalisation

c. resulting in persistent or significant disability / incapacity

d. life-threatening

e. involving malignancy or congenital anomaly.

Medical judgement should be exercised in deciding whether an adverse event/ reaction is serious in other situations. Important adverse events/ reactions that are not immediately life-threatening or do not result in death or hospitalisation but may jeopardise the patient or may require intervention to prevent one of the other outcomes listed in the definition above, should also be considered serious.

Starting from Visit 2, and for all subsequent examinations during the trial, all symptoms or diseases which were not present at the initial examination (Visit 1) must be recorded. Documentation regarding the adverse event should be made as to the nature, date of onset, end date, seriousness, severity, course, relation to trial medications, action(s) taken and outcome of any signs or symptoms observed by the physician or reported by the patient upon indirect questioning or recorded on a diary card. In any case the patient has to be followed carefully until the adverse event has resolved or at least stabilised. If there is a worsening of a medical condition that was present before starting the trial, this should be considered as an adverse event and a complete evaluation should be recorded. The trial medications relationship to each adverse event should be determined by the investigator based on the following definition:

8.3. DEFINITION 3

- Certain: A clinical event, including laboratory test abnormality, occurring in a plausible time relationship to drug administration, and which cannot be explained by concurrent disease or other drugs or chemicals. The event must be plausible pharmacologically or phenomenologically, and its clinical response to withdrawal of the drug (dechallenge) should be clinically plausible. It must also be confirmed by a satisfactory challenge procedure.

NOTE: The decision to perform a formal rechallenge must be made by the Investigator and the Clinical Project Manager after reviewing the patient's complete history.

- Probable: A clinical event, including laboratory test abnormality, with a reasonable time sequence to drug administration, unlikely to be attributed to concurrent disease or other drugs or chemicals, and which follows a clinical plausible response on withdrawal (dechallenge). Rechallenge information is not required to fulfil this definition.

- **Possible:** A clinical event, including laboratory test abnormality, with a reasonable time sequence to administration of the drug, but which could also be explained by concurrent disease or other drugs or chemicals. Information on drug withdrawal may be lacking or unclear.
- **Unlikely:** A clinical event, including laboratory test abnormality, with temporal relationship to drug administration which makes a causal relationship improbable, and in which other drugs, chemicals or underlying disease provide more plausible explanations.
- **Unassessable:** A report suggesting an adverse drug reaction, which cannot be judged because information is insufficient or contradictory, and which cannot be supplemented or verified.
- **Not related:** An adverse event, which is definitely not related causally to drug administration.

8.4. REPORTING AND RECORDING OF ADVERSE EVENTS

All symptoms or diseases observed by the physician or reported by the volunteer upon indirect questioning and which were not present at the initial examination (Visit 1) must be recorded as an adverse event.

Documentation regarding the adverse event should be made as to the nature, date of onset, end date, seriousness, severity, rate of appearance, corrective therapy, relation to trial medication, action(s) taken and outcome of any signs and symptoms.
In any case, the volunteer has to be followed carefully until the adverse event has resolved or at least stabilized. If there is a worsening of a medical condition that was present before starting the trial, this should be considered as an adverse event and a complete evaluation should be recorded.

The trial medications relationship to each adverse event should be determined by the investigator based on the definitions detailed above.
All information regarding adverse events must be recorded in the specific AE pages of the CRF.

8.5. REPORTING, RECORDING AND HANDLING OF SERIOUS ADVERSE EVENTS (SAES)

Any serious adverse event must be reported to benepharma within 24 hours.
All information regarding the experience must be recorded on a Serious Adverse Event (SAE) form. The patient should be followed carefully until the condition

disappears and/or the aetiology has been determined and all follow-up information has been obtained and recorded on the SAE form.

9 DATA MANAGEMENT AND STATISTICS

9.1. DATA MANAGEMENT

Data entry will be performed by the study site. The data will be entered by an authorized person onto a computer database.
All data entered onto the database will be checked again for accuracy and completeness using a trial specific computer-checking program. Any query resulting from this second check will be documented on a data correction form, answered by the investigator allowing correction of the database.

Programming of data checks, volunteer listings, summary tables and statistical tests will be performed by the Data Management and/or Statistician.

9.2. BLIND REVIEW

The ICH-Biostatistics Guideline E9 [31] recommends a blind review before the decoding process . This will be done for two purposes:

- To define data sets/populations with discussion of each problem case in a blinded manner.
- To check and eventually to amend the analysis plan, based on newly gained information in this domain of research and also based on first blinded data analyses.

After signing and dating meeting minutes the study may be unblinded concerning the type of study drug. A written confirmation of the database lock has to be issued by the responsible statistician, before code break.

9.3. SAMPLE SIZE CALCULATION

The sample size is difficult to calculate. In a previous thesis of our group sputum weight was found to be of little variation at two separate time points in patients with asthma. After inhalation of hypertonic saline induced sputum of 20 asthmatics was 4.9 $\pm$ 2.41 g and 4.78 $\pm$1.6 g respectively.
These data are is in keeping with a previous publication in chronic bronchitis with bronchiectasis. The authors found after inhalation of hypertonic a sputum weight of 5.3 g compared to 3.17 g after saline inhalation (24).
Therefore the calculated sample size of the present study is based on an expected difference for Tacholiquin ® in sputum weight of about 1.2 g corresponding to about 40% of that expected for saline with a standard deviation of s=1.7 g roughly

corresponding to a correlation between sputum weights of ρ=0.75. Then a sample size of 20 achieves a power of 80%.

9.4. ANALYSIS OF EFFICACY CRITERIA

The primary endpoint is sputum weight after 3 weeks of Tylaxopol– that after 3 weeks of saline treatment which will be analyzed for the Per Protocol Population. Estimated differences will be presented together with 95% confidence intervals (CIs) for the difference and two-sided p-values from a corresponding t-test. This analysis will be repeated for the ITT Population.

Secondary endpoints: The analysis of symptom scores ((CAT, BDI and TDI), ease of sputum production by analog scale, lung function parameter (FVC, FEV1, MEF25, RV, RV/TLC), will use the same methodology as that for the primary endpoint, using the ITT population. The analysis will include Day 1 and week 3 data. In addition acute changes of sputum weight on Day 1 at each treatment arm will be analyzed as described for the primary parameter. Secondary analyses aim to determine the efficacy BP versus placebo to modulate CRP and reducing the symptoms.

Data will be summarized with respect to demographic and baseline characteristics, efficacy observations and measurements, and safety observations and measurements.

Primary and secondary confirmatory analyses will be performed and reported at the end of the double-blind treatment period. A full statistical analysis plan will be produced prior to unblinding the study.

Biomarker profiles (sputum cells, IL-1, IL-6, IL-8), in sputum will be analyzed using an analysis of covariance (ANCOVA) model with covariates of baseline, smoking status and treatment. Estimated treatment differences along with corresponding 95 % CIs will be presented for the treatment comparison.

9.5. EXPLORATORY ANALYSIS OF EFFICACY CRITERIA

All statistical tests will be two-sided using alpha=0.05. An appropriate pooled center algorithm will be defined prior to unblinding the data. No adjustment for multiple testing will be done since the primary variable and the primary population is well defined.

9.6. ANALYSIS OF SAFETY DATA

The assessment of safety will be based mainly on the frequency of adverse events and on the laboratory data analysis detailed below. Vital signs will also be considered. .

The following analyses will be presented for laboratory data:

- Mean, median and change from baseline in laboratory values will be presented by visit.

9.7. ANALYSED POPULATION

The intent-to-treat population will be used for the primary and secondary analyses. Background data will be summarized for intent-to-treat patients. The per-protocol population will be used for the primary efficacy analysis only. Data from all patients will be listed.

10 ETHICAL CONSIDERATIONS AND PATIENT'S CONSENT

10.1. ETHICAL CONSIDERATIONS

The study will be carried out in accordance with the principles stated in the Declaration of Helsinki, last revised version (see Attachment 23.5) and in accordance with Good Clinical Practice (GCP) and applicable regulatory requirements.

10.2. ETHICS COMITTEES (EC), COMPETENT AUTHORITIES (CA)

According to the regulatory requirements of the country, the Sponsor will submit the study documentation to the relevant Ethics Committee (EC).

Patients must not be entered into the study until approval of the Ethics Committee and notification of Competent Authorities as defined by local regulatory requirements. All subsequent substantial amendments must be submitted to the Ethics Committee(s) for approval/information and/or to the Competent Authorities according to the local requirements.

The Ethics Committee(s) must also be informed of any serious and unexpected adverse reactions (SUSARs) occurring during the study, which are likely to affect the safety of the volunteers or the conduct of the study.

10.3. INFORMED CONSENT

The investigator must explain to each patient (or legally authorized representative) the nature of the study, its purpose, the procedures involved, the expected duration, the potential risks and benefits involved and any discomfort it may entail. Each patient must be informed that participation in the study is voluntary and that he/she may withdraw from the study at any time and that withdrawal of consent will not affect his/her subsequent medical treatment or relationship with the treating physician.

11 INSURANCE

During the trial an insurance will cover all patient risks and oligations which are necessary for a clinical trial.

12 PATIENT DOCUMENTATION AND MONITORING

12.1. CASE REPORT FORM (CRF)

CRFs for individual patients will be provided by the study site.

Patients are identified, according to national requirements, solely by the pre-printed CRF number and randomisation number. CRFs are used to record clinical trial data and are an integral part of the study and subsequent reports. The entries, therefore, must be legible and complete. The CRF can be completed by the investigator/authorized persons (mentioned in the centre study personnel identification form). Errors should be lined out but not obliterated and the correction inserted, initialed and dated.

13 DRUG ACCOUNTABILITY

The investigator must maintain accurate and adequate records including dates, batch number, quantities received, individual usage, etc. The investigator must also return to the Sponsor unused supplies giving an exact amount of usage in the study whether completed or terminated. At the time of return to the Sponsor, the investigator must verify that all unused or partially used drug supplies have been returned by the volunteer and that no remaining supplies are in his/her possession.

14 ADMINISTRATIVE ISSUES

14.1. RULES FOR AMENDING THE PROTOCOL

Any change to the protocol, once the final version has been issued, has to be detailed in a protocol amendment. Amendments to the trial are regarded as “substantial” where they are likely to have a significant impact on:

- the safety or physical or mental integrity of the subjects;
- the scientific value of the trial;
- the conduct or management of the trial;
- or the quality or safety of any investigational medicinal product (IMP) used in the trial.

In all cases, an amendment is only to be regarded as “substantial” when one or more of the above criteria is met. Substantial amendments must be submitted to the Competent Authorities (CA) and Ethics Committees concerned for approval. Non-substantial amendments have to be sent for notification to the CA and EC and should be available upon request for inspection at the trial site and/or Sponsor’s premises as appropriate.

All amendments must be numbered, dated and signed. Substantial amendments must be signed at least by the coordinating investigator and the Sponsor/CRO, non-substantial amendments will be signed by the Sponsor and/or CRO and the coordinating investigator, if appropriate.

14.2. ARCHIVING

The investigator must keep the study specific volunteer documentation for at least 10 years after termination of the trial. The investigator should take measures to prevent accidental or premature destruction of the study documents as long as they have to be maintained according to the applicable regulatory requirements and at least until the Sponsor has informed him/her that they no longer need to be retained.

15 STATEMENT OF CONFIDENTIALITY

Individual volunteer medical information obtained as a result of this study is considered confidential and disclosure to third parties is prohibited. Subject confidentiality will be further ensured by utilising subject identification code numbers to correspond to volunteer data in the computer files.

Data generated as a result of this study are to be made available for inspection/audit on request of the Sponsor, the Ethics Committee and/or the Competent Authorities.

16 SECRECY AGREEMENT

The investigator commits him/herself to keep secret from third parties any confidential information obtained from and concerning benepharma or this company's products, which in connection with the present contractual relationship are made available or disclosed, respectively, and to use this knowledge only as agreed upon. This commitment is valid independently of the existence and the duration of the present actual relationship, but only so far and so long as benepharma is reasonably and justly interested in the investigator’s maintaining this secrecy undertaking.

17 REPORTING AND PUBLICATION

Any formal presentation or publication of data from this trial will be considered as a joint publication by the investigator(s) and appropriate benepharma personnel. Authorship will be determined by mutual agreement.

18 FINANCIALS

Financials are listed in a separate document.

19 CONTINUATION SUPPLY

After completion of the trial no continuation supplies will be made available for the patients. If a treatment is necessary the investigator will treat the patients according to the science based guidelines. Alternatively, the investigator will inform the patient’s general practitioner (GP) at the end of the trial period. The patient will then be advised to visit his GP directly.

20 SCIENTIFIC AGREEMENT / SIGNATURES

20.1. AGREEMENT

The protocol should be signed and dated at least by the Study Director and the sponsor benepharma.

20.2. SIGNATURES

By signing the document, the agrees to conduct the study as outlined in the above protocol including attachments, which contains all details necessary for carrying out the study. The investigation will be completed within the agreed timelines.

Prof. Dr. med. Stefan Zielen
Klinik für Kinder- und Jugendmedizin
Allergologie, Pneumologie und Mukoviszidose
Goethe-Universität
Theodor-Stern-Kai 7
60590 Frankfurt/Main

Date Signature

Dr. med. Gerald Untersteiner
bene-Arzneimittel GmbH
Herterichstr. 1-3
D - 81479 München
T +49 89 74 987 - 232
F +49 89 74 987 – 233
E-Mail:g.untersteiner@bene-gmbh.de

Date Signature

21 REFERENCES

1. Vesbo J, Hogg JC. Convergence of the epidemiology and pathology of COPD. Thorax 2006; 61: 86–88.
2. Brumfitt W, Willoghby MLN, Bromley LL. An evaluation of sputum examination in chronic bronchitis. Lancet 1957; 273: 1306–1309.
3. Thurlbeck WM, Angus GE. The relationship between chronic bronchitis and emphysema as assessed morphologically. Am Rev Respir Dis 1963; 87: 815–819.
4. Vestbo J, Prescott E, Lange P. Association of chronic mucus hypersecretion with FEV1 decline and chronic obstructive pulmonary disease morbidity. Copenhagen City Heart Study Group.
   Am J Respir Crit Care Med 1996; 153: 1530–1535.
5. de Marco R, Accordini S, Cerveri I, et al. Incidence of chronic obstructive pulmonary disease in a cohort of young adults according to the presence of chronic cough and phlegm. Am J Respir Crit Care Med 2007; 175: 32–39.
6. Kohansal R, Martinez-Camblor P, Agusti A, et al. The natural history of chronic airflow obstruction revisited: an analysis of the Framingham offspring cohort. Am J Respir Crit Care Med 2009; 180:3–10.
7. Decramer M, Janssens W. Mucoactive therapy in COPD. Eur Respir Rev 2010; 19: 116, 134–140.
8. Balsamo R, Lanata L, Egan CG. Mucoactive drugs. Eur Respir Rev 2010; 19: 127–133.
9. Hutschenreuter: Die Bedeutung moderner Netzmittel für die Chirurgie. Der Chirurg, 1958; 29: 6, 252-258.
10. Wageneder: Tacholiquin in der prä- und postoperativen Phase bei thoraxchirurgischen Eingriffen Münchner Med. Wochenschr 1962; 47, 2296-2298.
11. Stieve: Netzmittel in der Aerosoltherapie. Zeitschr. für Aerosolforsch. u. –therapie, 1957; 6: 3, 215-244.
12. Chowanetz, Denck: Die Vorteile der Tacholiquin-Aerosolbehandlung in der Chirurgie Wiener Med. Wochenschr. 1962;112 : 43, 818-819
13. Palmer KN.: A new mucolytic agent by aerosol for inhalation in chronic bronchitis Lancet 1961: 7;802-804
14. Kittelmann B, Vogel J, Lachmann B, Wuthe H. Comparison of lung-mechanical and physico-chemical Parameters in view of the pulmonary surfactant system. Z Erkr Atmungsorgane. 1976; 145(3):308-16.
15. Ravvenel SF. New technique of humidification in pediatrics. J Am Med Assoc. 1953; 28;151(9):707-11.

16. Conley JJ. Diagnosis and treatment of encrustations in the trachea: their relation to radical surgery of the head and neck. J Am Med Assoc. 1954; 6;154(10):829-32.
17. Tainter ML, Nachod FC, Bird JG. Alevaire as a mycolytic agent. N Engl J Med. 1955: 3;253(18):764-7.
18. Wilde A. Oberflächenspannungs- und Viskositäts-Messungen an Sputa unter dem Einfluss von Tacholiquin. Notabene Medici, 1978: 8. Heft 1.
19. Paez PN, Miller WF.Surface active agents in sputum evacuation: a blind comparison with normal saline solution and distilled water. Chest. 1971;60(4):312-7.
20. Farber SM, Benioff MA, Smith JD. Planned care for patients with bronchiectasis Calif. Med. 1955: 82; 2:102-106,
21. Ghio AJ, Marshall BC, Diaz JL, Hasegawa T, Samuelson W, Povia D, Kennedy TP, Piantodosi CA. Tyloxapol inhibits NF-kappa B and cytokine release, scavenges HOCI, and reduces viscosity of cystic fibrosis sputum. Am J Respir Crit Care Med. 1996; 154(3 Pt 1):783-8.
22. Miller JB, Boyer EH. A nontoxic detergent for aerosol use in dissolving viscid bronchopulmonary secretions. J Pediatr. 1952 ;40(6):767-71
23. Segal MS, Attinger E. Advances in inhalational therapy in the management of diseases of the chest. Am J Surg. 1955;89(2):387-401
24. Camarata SJ, Jacobs HJ, Affeldt JE. The use of enzymes and wetting agents in the treatment of pulmonary atelectasis. Dis Chest. 1956 ;29(4):388-401.
25. Eickmeier O, Huebner M, Herrmann E, Zissler U, Rosewich M, Baer PC, Buhl R, Schmitt-Grohé S, Zielen S, Schubert R. Sputum biomarker profiles in cystic fibrosis (CF) and chronic obstructive pulmonary disease (COPD) and association between pulmonary function. Cytokine. 2010 ; 50(2):152-7.
26. Kellett F, Redfern J, Niven RM Evaluation of nebulised hypertonic saline (7%) as an adjunct to physiotherapy in patients with stable bronchiectasis. Respir Med. 2005 ;99 (1):27-31.
